# Integrated network reconstruction, visualization and analysis using YANAsquare

**DOI:** 10.1186/1471-2105-8-313

**Published:** 2007-08-28

**Authors:** Roland Schwarz, Chunguang Liang, Christoph Kaleta, Mark Kühnel, Eik Hoffmann, Sergei Kuznetsov, Michael Hecker, Gareth Griffiths, Stefan Schuster, Thomas Dandekar

**Affiliations:** 1 Department of Bioinformatics, Biocenter Am Hubland, D-97074 University of Würzburg, Germany; 2 Bio Systems Analysis Group, Department of Mathematics and Computer Science, Friedrich Schiller University Jena, Germany; 3 Structural and Computational Biology, EMBL Heidelberg, Germany; 4 Department of Cell Biology and Biosystems Technology, Albert-Einstein-Str. 3, D-18059 University of Rostock, Germany; 5 Institute for Microbiology, Friedrich-Ludwig-Jahn-Str. 15, D-17487 University of Greifswald, Germany; 6 Department of Bioinformatics, Ernst-Abbe-Platz 2, D-07743 University of Jena, Germany; 7 Cell Biology Program, EMBL Heidelberg, Germany

## Abstract

**Background:**

Modeling of metabolic networks includes tasks such as network assembly, network overview, calculation of metabolic fluxes and testing the robustness of the network.

**Results:**

YANAsquare provides a software framework for rapid network assembly (flexible pathway browser with local or remote operation mode), network overview (visualization routine and YANAsquare editor) and network performance analysis (calculation of flux modes as well as target and robustness tests). YANAsquare comes as an easy-to-setup program package in Java. It is fully compatible and integrates the programs YANA (translation of gene expression values into flux distributions, metabolite network dissection) and Metatool (elementary mode calculation). As application examples we set-up and model the phospholipid network in the phagosome and genome-scale metabolic maps of *S.aureus*, *S.epidermidis *and *S.saprophyticus *as well as test their robustness against enzyme impairment.

**Conclusion:**

YANAsquare is an application software for rapid setup, visualization and analysis of small, larger and genome-scale metabolic networks.

## Background

When studying medium-sized to large metabolic networks, topological and structural analyses are often the only feasible approach to analyze them, as the required kinetic parameters of enzymes and needed mechanistic details of the underlying reactions are rarely available. Steady-state analyses [1] such as Elementary Mode Analysis (EMA) [2], the associated theory of extreme pathways [3] and Flux Balance Analysis (FBA) [4], have proven especially useful to study such networks with respect to e.g. minimal cutsets [5] for drug target identification [6,7] and robustness analysis [8,9] or estimation of maximal metabolite yield [10].

The algorithm for elementary mode analysis has been implemented (e.g. Metatool [11,12]) and integrated into a number of tools for pathway analysis such as GEPASI [13] or SNA [14].

We introduced YANA [15] which integrates the field-tested Metatool program into a coherent user-friendly interface with several additional useful algorithms for the steady-state analysis of metabolic networks. Applying a genetic algorithm, it links gene expression data to estimated flux distributions and vice versa. Different approaches for such a calculation and fit have been proposed [16], YANA includes a particular robust minimization strategy which minimizes the error for both fits even if noisy data or very few measurements are given [15].

In several interdisciplinary projects we recently combined *in-silico *pathway analysis and prediction of flux distributions with experimental measurements, such as ^13^C isotopologue measurements on *Listeria monocytogenes *[17]. With our modeling platform we achieve in the present study the rapid setup and analysis of large metabolic networks by combining (i) a direct Java implementation of the elementary mode algorithm for increased platform independence and performance; (ii) a module for the rapid automatic set-up of genome-wide metabolic networks by direct access to the Kyoto Encyclopedia of Genes and Genomes (KEGG) [18] including a smart editor for reworking of the results; (iii) algorithms for the visualization and graphical analysis of metabolic networks, including automatic layout routines and (iv) systematic robustness analysis for testing the network's stability towards enzyme deletions.

Here a discussion on the limitations of EMA is appropriate. Our software allows rapid set-up of the network of interest. However, the choice of external and internal metabolites by the user has a strong impact on the results of EMA. In general, for large networks also a strong pre-knowledge on how to decompose very large networks based on biochemical constraints is needed, otherwise the results are misleading. Similarly, a combinatorial explosion of modes can often be reduced or even avoided by a careful choice of the form of networks and sub-networks. The tutorial in the supplementary material gives for this a number of hints and rules but is of course no substitute for an expert.

As application examples we rapidly set-up and compare genome-scale metabolic networks of different *Staphylococci*, test systematically the systems' robustness against gene deletions as well as analyze and extend complex phospholipid networks in the murine phagosome.

## Implementation

### Steady state analysis

To increase platform independence and avoid loss of computation time (e.g. by parsing of the Metatool output) we included a Java implementation of the well known Schuster algorithm [2] which computes the EMs through a step-wise satisfaction of the steady state condition for each metabolite. The original version of the algorithm has been improved according to Klamt and Gagneur 2004 [19] by representing EMs during calculation by bit patterns rather than by their fluxes. This is possible due to the existence of a direct mapping of the set of reactions of an EM to the fluxes of these reactions [19]. The most frequently called function during the computation, the test for elementarity of an intermediate EM, is then reduced to a mere bit operation which drastically improves the algorithm's runtime behavior. This implementation is also used by a software package destinated at the computation of chemical organizations in chemical reaction networks [20].

The current version allows computation of the complete set of EMs or only the convex basis, both using either the external Metatool or the newly implemented internal EMA routine. We tested the algorithm thoroughly for consistency with the Metatool results.

### KEGG Browser (KGB)

Successful analysis of a metabolic network which is expected to give biologically meaningful results heavily depends upon its accurate reconstruction. Complete sets of all modeled metabolites and enzymes have to be set up, and system boundaries have to be defined carefully. Every enzyme has to be checked for its absence or presence in the actual organism by investigation of genome annotations, homology searches or literature data mining. This task is hindered additionally by the missing or only partially established standardization of compound and enzyme names. Public databases such as Brenda [21], Enzyme [22] or the KEGG encyclopedia [18] have eased this process considerably by providing algorithms and interfaces to find the correct subset of enzymes to work on, their associated pathway structures and check for their occurrences in the organism under study. They further aim to unify the different available naming conventions, but so far the actual network setup in the modeling software had to be done manually. To further accelerate and automate the initial setup of a metabolic network we implemented the KEGG Browser (KGB) module in YANAsquare. It is capable of connecting directly to the KEGG database to browse pathways, reaction lists, metabolites and pathway maps online. Organisms or reference pathways can be selected to only retrieve enzymes which have been annotated as present in the selected organism or to obtain the complete set of available reactions respectively. All associated metabolites are collected automatically, highly abundant metabolites such as H^+^, CO^2^, H_2_O, ADP or ATP considered to be well buffered can be filtered out to lower the network complexity in the EMA analysis.

One problem when dealing directly with the KEGG database entries is that metabolites and reaction names are often long and may contain special characters. For a later visualization of the network, for backwards-compatibility to the Metatool input files and most importantly to satisfy the regular expression facets of the SBML2 standard which serves as our main file format, identifiers for enzymes and metabolites have to be standardized. We implemented an automatic abbreviation routine capable of shortening chemical names in a standardized way using a dictionary of keyword abbreviations. Generated abbreviations are validated so that characters illegal in the context of the SBML2 or Metatool format definitions will be eliminated. As enzyme names from ExPASy are generally even shorter, an additional step for the routine is to adapt shorter alternative names from ExPASy, which can again be further shortened by the abbreviation approach if necessary.

When accessing KEGG through a low-bandwidth Internet connection the retrieval of large chunks of reactions can be time consuming. We therefore additionally provide an accelerated query method using a local reaction cache (instead of retrieving all records directly via SOAP). This semi-online mode works significantly faster but the reaction cache has to be updated on a regular basis to assure that the KGB makes use of the latest KEGG definitions and annotations, an operation which can easily be performed through the KEGG Browser's update manager.

Once the desired topology has been assembled, the chosen reactions and metabolites can be imported directly into YANAsquare for further network analysis.

**Figure 1:**
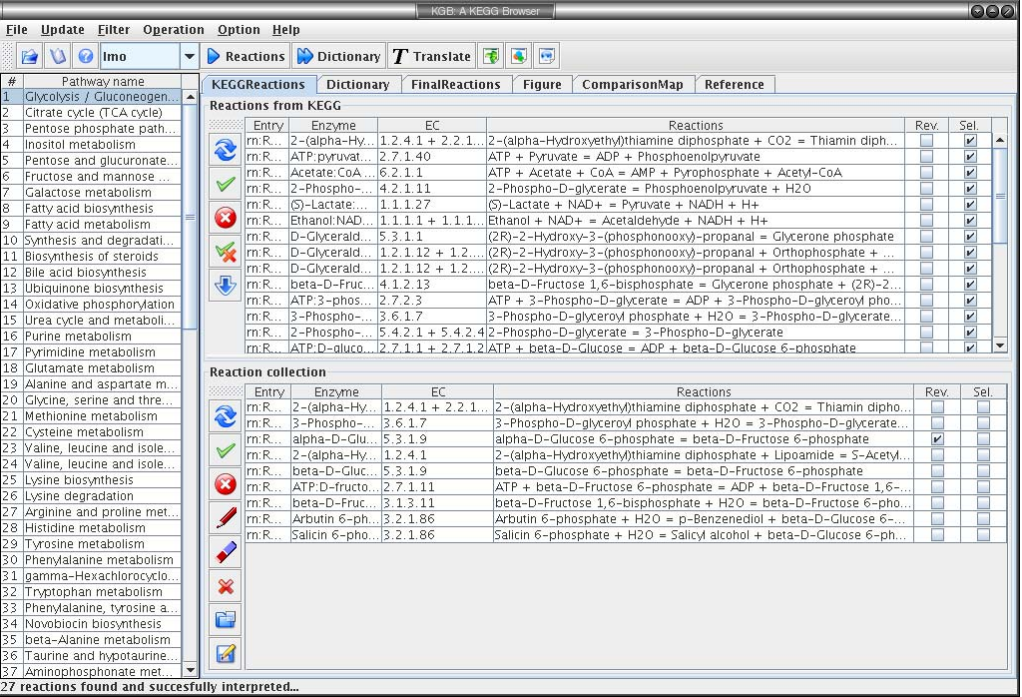
**The KEGG Browser module in YANAsquare**. Screenshot of the KGB KEGG Browser extension to YANAsquare. For a pathway selected from the list of KEGG pathways to the left the browser shows all corresponding reactions in tabular form or as the original metabolic map.

We used version 1.3 of the Apache Axis library, a Java implementation of the Simple Object Access Protocol (SOAP) and Web Services Description Language (WSDL) on top of the HTTP protocol to connect to the database server. We developed a user-friendly graphical interface using the SWING framework to retrieve the information from KEGG (see Fig. 1). Pathways and reactions of interests are specified by the user, all reaction equations are retrieved and listed in a table. A smart editor allows users to edit the results, change metabolite and enzyme names and abbreviations and/or append novel reactions if these are known to the user but not yet reported in KEGG. The whole software can also be easily adopted to extract data from another database, if format requirements are adapted or met, e.g. a private in-house metabolite and enzyme database in the KEGG format.

### Visualization

With increasing network complexity the results from a steady-state analysis suffer from the combinatorial explosion of elementary modes. A realistic system comprising of about 100 metabolites and enzymes can easily result in over 100, 000 elementary modes. Interpretation of these data is close to infeasible when the results from the analysis are retrieved in form of a tabular listing of all elementary modes. Typical questions asked here are how the flux distribution for a specific set of active pathways is distributed across the network, which parts of the network are affected by certain sets of elementary modes or how deactivation of a set of pathways changes the overall network flux.

**Figure 2:**
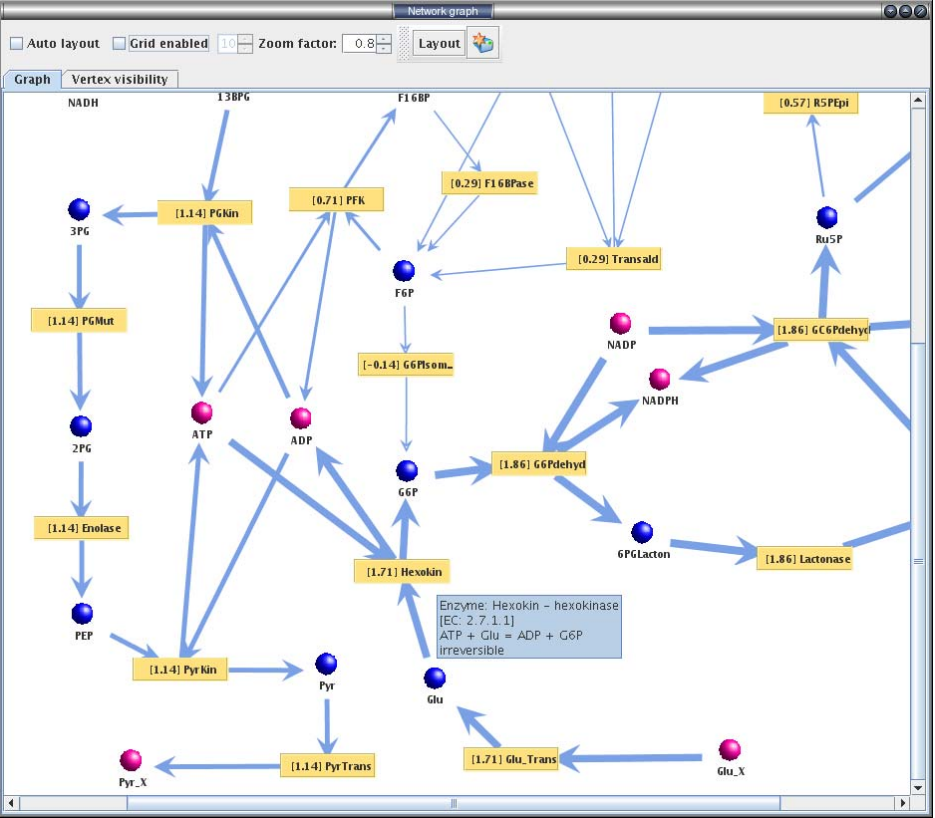
**Metabolic network visualization using YANAsquare**. Visualization of a metabolic network as a bipartite graph using YANAsquare. Metabolites are drawn as bullets where blue indicates internal (or balanced) metabolites and purple bullets depict metabolites outside system boundaries (external metabolites). Enzymes are drawn as yellow squares including the flux through the enzyme given the current set of elementary mode activities. Tooltip texts give detailed information about both enzymes and metabolites such as net reaction, reversibility and enzyme description. The widths of the arrows give a quick indication of the relative amount of flux through the reaction compared to the overall flux distribution.

To circumvent these difficulties we implemented a visualization routine in YANAsquare capable of displaying the metabolic network as a bipartite graph, where the two vertex classes correspond to reactions and metabolites, respectively. We added several field-tested layout algorithms (e.g. spring-embedded layout, radial tree, sugiyama) to automatically arrange the nodes and edges of the network in a user-friendly way. While this visualization greatly facilitates the setup of metabolic networks, it is especially useful after performing a steady-state analysis of the network: YANAsquare is capable of projecting the flux distribution resulting from the set of EM activities onto the network graph, displaying the net flux through a given enzyme next to its vertex and scaling the width of the graph edges accordingly (smaller or bigger blue arrows in Fig. 2). This gives the user the possibility to directly inspect the network graphically for flux bottlenecks, hub or choke points and to see the overall flux distribution across the network at a glance. Individual fluxes through one enzyme for specific modes can also be analyzed and visualized.

### Robustness analysis

Experiments in pharmacology, infection biology or genetics aim to modify or eliminate enzymes to study network stability. To further add to YANA's network analysis capabilities for such questions, a robustness test algorithm was implemented, elaborating on earlier ideas [9]. For a stoichiometric *m × n *matrix *S *with *m *metabolites and *n *enzymes, a matrix of elementary modes *E *(*n × e*) of *e *elementary flux modes and a vector of flux mode activities *v *the vector of metabolite productions

*p *= *SEv*

is computed. The algorithm iterates over the enzymes and deactivates all pathways which use the enzyme under question (setting the respective components of *v *to zero), leading to *n *vectors of elementary flux mode activities *v*_*i*_, *i *∈ 1..*n*. After each step the number of external metabolites which can still be produced by the system is computed into an overall vector of average production

p(avg)=1n∑i=1nℋ(SEvi)
 MathType@MTEF@5@5@+=feaafiart1ev1aaatCvAUfKttLearuWrP9MDH5MBPbIqV92AaeXatLxBI9gBaebbnrfifHhDYfgasaacH8akY=wiFfYdH8Gipec8Eeeu0xXdbba9frFj0=OqFfea0dXdd9vqai=hGuQ8kuc9pgc9s8qqaq=dirpe0xb9q8qiLsFr0=vr0=vr0dc8meaabaqaciaacaGaaeqabaqabeGadaaakeaacqWGWbaCdaahaaWcbeqaaiabcIcaOiabdggaHjabdAha2jabdEgaNjabcMcaPaaakiabg2da9maalaaabaGaeGymaedabaGaemOBa4gaamaaqahabaWenfgDOvwBHrxAJfwnHbqeg0uy0HwzTfgDPnwy1aaceaGae83cHGKaeiikaGIaem4uamLaemyrauKaemODay3aaSbaaSqaaiabdMgaPbqabaGccqGGPaqkaSqaaiabdMgaPjabg2da9iabigdaXaqaaiabd6gaUbqdcqGHris5aaaa@5028@

of metabolites, where ℋ
 MathType@MTEF@5@5@+=feaafiart1ev1aaatCvAUfKttLearuWrP9MDH5MBPbIqV92AaeXatLxBI9gBaebbnrfifHhDYfgasaacH8akY=wiFfYdH8Gipec8Eeeu0xXdbba9frFj0=OqFfea0dXdd9vqai=hGuQ8kuc9pgc9s8qqaq=dirpe0xb9q8qiLsFr0=vr0=vr0dc8meaabaqaciaacaGaaeqabaqabeGadaaakeaat0uy0HwzTfgDPnwy1egaryqtHrhAL1wy0L2yHvdaiqaacqWFlecsaaa@3762@(*x*) denotes the Heaviside function

ℋ(x)={1if x>00otherwise
 MathType@MTEF@5@5@+=feaafiart1ev1aaatCvAUfKttLearuWrP9MDH5MBPbIqV92AaeXatLxBI9gBaebbnrfifHhDYfgasaacH8akY=wiFfYdH8Gipec8Eeeu0xXdbba9frFj0=OqFfea0dXdd9vqai=hGuQ8kuc9pgc9s8qqaq=dirpe0xb9q8qiLsFr0=vr0=vr0dc8meaabaqaciaacaGaaeqabaqabeGadaaakeaat0uy0HwzTfgDPnwy1egaryqtHrhAL1wy0L2yHvdaiqaacqWFlecscqGGOaakcqWG4baEcqGGPaqkcqGH9aqpdaGabeqaauaabaqaciaaaeaacqaIXaqmaeaacqqGPbqAcqqGMbGzcqqGGaaicqWG4baEcqGH+aGpcqaIWaamaeaacqaIWaamaeaacqqGVbWBcqqG0baDcqqGObaAcqqGLbqzcqqGYbGCcqqG3bWDcqqGPbqAcqqGZbWCcqqGLbqzaaaacaGL7baaaaa@51F0@

and is here defined component-wise.

In the same way we define the total metabolite production vector

p(tot)=ℋ(SE1)
 MathType@MTEF@5@5@+=feaafiart1ev1aaatCvAUfKttLearuWrP9MDH5MBPbIqV92AaeXatLxBI9gBaebbnrfifHhDYfgasaacH8akY=wiFfYdH8Gipec8Eeeu0xXdbba9frFj0=OqFfea0dXdd9vqai=hGuQ8kuc9pgc9s8qqaq=dirpe0xb9q8qiLsFr0=vr0=vr0dc8meaabaqaciaacaGaaeqabaqabeGadaaakeaacqWGWbaCdaahaaWcbeqaaiabcIcaOiabdsha0jabd+gaVjabdsha0jabcMcaPaaakiabg2da9mrtHrhAL1wy0L2yHvtyaeHbnfgDOvwBHrxAJfwnaGabaiab=TqiijabcIcaOiabdofatjabdweafHqabiab+fdaXiabcMcaPaaa@44E7@

of the unrestricted network (**1 **denotes the vector of all ones). The total production indicates what the system produces if no enzyme is impaired. The average production indicates the average system production after knock-out of one enzyme. By summing up over the components of the vectors we get the (scalar) total and average production sums *s*^(*tot*) ^and *s*^(*avg*) ^as well as the loss *l*

s(avg)=∑j=1mpj(avg)s(tot)=∑j=1mpj(tot)l=s(tot)−s(avg).
 MathType@MTEF@5@5@+=feaafiart1ev1aaatCvAUfKttLearuWrP9MDH5MBPbIqV92AaeXatLxBI9gBaebbnrfifHhDYfgasaacH8akY=wiFfYdH8Gipec8Eeeu0xXdbba9frFj0=OqFfea0dXdd9vqai=hGuQ8kuc9pgc9s8qqaq=dirpe0xb9q8qiLsFr0=vr0=vr0dc8meaabaqaciaacaGaaeqabaqabeGadaaakeaafaqadeWabaaabaGaem4Cam3aaWbaaSqabeaacqGGOaakcqWGHbqycqWG2bGDcqWGNbWzcqGGPaqkaaGccqGH9aqpdaaeWbqaaiabdchaWnaaDaaaleaacqWGQbGAaeaacqGGOaakcqWGHbqycqWG2bGDcqWGNbWzcqGGPaqkaaaabaGaemOAaOMaeyypa0JaeGymaedabaGaemyBa0ganiabggHiLdaakeaacqWGZbWCdaahaaWcbeqaaiabcIcaOiabdsha0jabd+gaVjabdsha0jabcMcaPaaakiabg2da9maaqahabaGaemiCaa3aa0baaSqaaiabdQgaQbqaaiabcIcaOiabdsha0jabd+gaVjabdsha0jabcMcaPaaaaeaacqWGQbGAcqGH9aqpcqaIXaqmaeaacqWGTbqBa0GaeyyeIuoaaOqaaiabdYgaSjabg2da9iabdohaZnaaCaaaleqabaGaeiikaGIaemiDaqNaem4Ba8MaemiDaqNaeiykaKcaaOGaeyOeI0Iaem4Cam3aaWbaaSqabeaacqGGOaakcqWGHbqycqWG2bGDcqWGNbWzcqGGPaqkaaGccqGGUaGlaaaaaa@70B9@

The odds ratio

r=s(avg)s(tot)
 MathType@MTEF@5@5@+=feaafiart1ev1aaatCvAUfKttLearuWrP9MDH5MBPbIqV92AaeXatLxBI9gBaebbnrfifHhDYfgasaacH8akY=wiFfYdH8Gipec8Eeeu0xXdbba9frFj0=OqFfea0dXdd9vqai=hGuQ8kuc9pgc9s8qqaq=dirpe0xb9q8qiLsFr0=vr0=vr0dc8meaabaqaciaacaGaaeqabaqabeGadaaakeaacqWGYbGCcqGH9aqpdaWcaaqaaiabdohaZnaaCaaaleqabaGaeiikaGIaemyyaeMaemODayNaem4zaCMaeiykaKcaaaGcbaGaem4Cam3aaWbaaSqabeaacqGGOaakcqWG0baDcqWGVbWBcqWG0baDcqGGPaqkaaaaaaaa@3E35@

expressed in percent is finally referred to as the overall robustness score of the network against single enzyme deletions.

Additionally, from the average activity of elementary modes

v(avg)=1n∑i=1nvi
 MathType@MTEF@5@5@+=feaafiart1ev1aaatCvAUfKttLearuWrP9MDH5MBPbIqV92AaeXatLxBI9gBaebbnrfifHhDYfgasaacH8akY=wiFfYdH8Gipec8Eeeu0xXdbba9frFj0=OqFfea0dXdd9vqai=hGuQ8kuc9pgc9s8qqaq=dirpe0xb9q8qiLsFr0=vr0=vr0dc8meaabaqaciaacaGaaeqabaqabeGadaaakeaacqWG2bGDdaahaaWcbeqaaiabcIcaOiabdggaHjabdAha2jabdEgaNjabcMcaPaaakiabg2da9maalaaabaGaeGymaedabaGaemOBa4gaamaaqahabaGaemODay3aaSbaaSqaaiabdMgaPbqabaaabaGaemyAaKMaeyypa0JaeGymaedabaGaemOBa4ganiabggHiLdaaaa@4175@

we compute the average number of active modes after a single gene knock-out

t=∑i=1evi(avg).
 MathType@MTEF@5@5@+=feaafiart1ev1aaatCvAUfKttLearuWrP9MDH5MBPbIqV92AaeXatLxBI9gBaebbnrfifHhDYfgasaacH8akY=wiFfYdH8Gipec8Eeeu0xXdbba9frFj0=OqFfea0dXdd9vqai=hGuQ8kuc9pgc9s8qqaq=dirpe0xb9q8qiLsFr0=vr0=vr0dc8meaabaqaciaacaGaaeqabaqabeGadaaakeaacqWG0baDcqGH9aqpdaaeWbqaaiabdAha2naaDaaaleaacqWGPbqAaeaacqGGOaakcqWGHbqycqWG2bGDcqWGNbWzcqGGPaqkaaaabaGaemyAaKMaeyypa0JaeGymaedabaGaemyzauganiabggHiLdGccqGGUaGlaaa@3FB2@

The average mode loss *m *is then given via

*m *= *e *- *t*

and returned as a result of the robustness analysis (see Table 1).

**Table 1:** Robustness analysis of three *Staphylococci *species

Organism	*S. aureus*	*S. epidermidis*	*S. saprophyticus*
Avg # of products	20.64	20.76	21.35
Robustness score	85.99%	86.51%	92.84%

The values show robustness differences among three *Staphylococci *species when glucose is supplied as the carbon source. The first row shows the average number (#) of metabolites produced, the second row is the robustness score *r*.

## Results

### Rapid network setup

A first example of the murine phospholipid network involved in phagosomal signaling illustrates the setup of a complex metabolic network of interest using YANAsquare. By querying the KEGG pathways "Glycerolipid metabolism (map 00561)", "Glycerophospholipid metabolism (map 00564)" and "Inositol phosphate metabolism (00562)" the complete set of 56 reactions and 64 metabolites available in the mouse metabolism is set up. After sensible setting of the system boundaries this leads directly to 57 elementary modes [see Tab. 4–6, Additional File 1]. Next, typical network extensions and modifications can be analyzed and illustrated. For instance, a reduced network with just 35 metabolites and 24 reactions is more accurate to identify key phospholipid conversions in the phagosome [see Tab. 1–3, Additional File 1]. In particular this reduced network includes as external components a number of phospholipid components which in the meantime have been shown to stimulate or inhibit the phagosome [23].

In this well connected network different conditions can be tested and interesting modes be rapidly visualized applying YANAsquare (as in Fig. 2), e.g. those modes yielding a specific phospholipid or those yielding ATP. Thus, setting ATP as an internal metabolite results in 14 ATP producing actin nucleation modes (from a total of 128 in this network) involving different phospholipids [for network definition and results, see Additional File 1]. Certainly this is a simplistic picture of the much more complex processes in phagosomal signaling. However the prediction of ATP generating actin nucleating modes identified and motivated experiments for different phospholipids, which are in fact in line with the model predictions. In particular, the modes suggest that the phospholipids phosphatidyl-inositol-phosphate and -bisphosphate (PIP and PIP2) as well as sphingosine-1-phosphate (S1P) stimulate the network whereas diacylglycerol (DAG) and phosphatidyl-choline (PC) are not in any of the modes and predicted to be inhibitory. This is in line with experimental data (Kühnel et al., unpublished, [23]) regarding exactly these phospholipids which have been obtained from phagosome actin nucleation assays. In these assays [23], murine phagosomes nucleate GFP labeled actin after phospholipid stimulation and the polymerization is monitored by confocal light microscopy.

Applying the KEGG browser additional phospholipid conversions involving e.g. phosphatidylethanolamine and phosphatidylsphingosine can easily be added. Furthermore, the network can be easily extended to prostaglandines and their conversions [KEGG map No. 00600, 00590, for network definition and results, see Additional File 1].

### Genome-scale pathway analysis in Staphylococci

The capabilities of YANAsquare further allow rapid and comparative genome-scale metabolic analyses. The tutorial and examples included in the supplementary material contain helpful hints for the user to achieve this, including network simplification rules to avoid combinatorial explosion of elementary modes. In our illustration example, different *Staphylococci *strains are compared for their main metabolic capabilities: *S. aureus COL*, *S. aureus N315*, *S. saprophyticus *and *S. epidermidis*. Note that the comparisons serve illustration purposes and more detailed studies are required for an accurate modeling of these strains. However the examples show how the tool YANAsquare allows a rapid first overview and points out metabolic differences.

The KEGG browser allows easy establishment of the complete network of interest. Regarding the growth equation of *S. aureus *[24], it is evident that we require the metabolism of central sugars, amino acids, and fatty acids as well as the nucleotide metabolism. This is easily compiled using the KEGG browser. Two different strains are readily accessed using KEGG: *S. aureus *COL and *S. aureus *N315. The central metabolism of *S. aureus *COL including carbohydrates, nucleotides, amino acids and lipid metabolism is rapidly established applying YANAsquare. Using the KEGG maps 00010, 00020, 00030, 00251, 00252, 00260, 00271, 00272, 00290, 00300, 00330, 00340, 00400, 00620, 00720, a system with 134 metabolites and 3957 modes is obtained [see Additional File 1, Tab. 10–15; for rules for abbreviation and network simplification see tutorial]. YANAsquare shows also quickly that regarding central metabolism, strain N315 is rather similar to *S. aureus *COL (same number of modes and metabolites). Clear differences in central metabolism become readily apparent changing to *S. epidermidis *and *S. saprophyticus *instead. Reading in the KEGG maps (the serial numbers are the same as in *S. aureus*) we now obtain a system with 134 metabolites and 11910 modes (*S. epidermidis*) as well as 132 metabolites and 11844 modes (*S. saprophyticus*), respectively [for example modes see Additional File 1; the complete list of modes is available from the authors on request].

A first comparison of the network models obtained predicts that *S. aureus *has lower ATP production than *S. epidermidis*, since its tricarboxylic acid cycle (TCA) is not complete: one involved enzyme – malate dehydrogenase (MDH; EC: 1.1.1.37) – is absent in *S. aureus *strains. It is a well-known NAD-dependent enzyme, which synthesizes oxaloacetate and NADH, the latter metabolite will contribute 3 moles of ATP through oxidative phosphorylation. A second enzyme, membrane-associated malate: quinone-oxidoreductase (EC: 1.1.99.16) can be expressed at certain stages. As this is then highly expressed in *S. aureus*, this partially compensates for the lack of MDH. However, the corresponding reaction requires external supply of Vitamin K (the *S. aureus *strain lacks the capability of producing it). The model for this can be readily established, e.g. by switching the menaquinol and menaquinone metabolites from internal to external in the software to simulate the synthesis of metabolites if Vitamin K is present in the medium. Comparing the resulting pathways between the different strains further, we find that the *S. aureus *strain can bypass the above TCA gap via malic enzyme (EC: 1.1.1.38), phosphoenolpyruvate-protein phosphotransferase (EC: 2.7.3.9) and phosphoenolpyruvate carboxylase (EC: 4.1.1.49) to produce oxaloacetate which can take part in the next round of the TCA cycle or be used to synthesize amino acids. However, this alternative way consumes ATP and requires the energy-consuming PTS system (EC: 2.7.1.69, EC: 2.7.3.9).

*S. aureus *has more functional enzymes and modes regarding amino acid production, whereas *S. saprophyticus *has fewer modes for certain amino acids. For instance, the absence of threonine aldolase (EC 4.1.2.5) disrupts the connection between threonine and glycine pathways. Even its urea cycle is not complete (e.g., *S. saprophyticus *modes no: 4 *- *8 etc.), as for instance carbamoyl phosphokinase (EC: 2.7.2.2) is not present. *S. saprophyticus *lacks the capability of producing carbamoyl phosphate directly. Instead, this is supplied from amino acids (e.g., *S. saprophyticus *modes no: 4 *- *8 etc.). All missing metabolic capabilities (e.g., compared to the modes no. 3, 19, 21, 81, 91, 100 in *S. aureus*) should potentially reduce the robustness, even though the system can still synthesize all the essential amino acids with an appropriate medium. *S. epidermidis *lacks arginase (EC: 3.5.3.1) and its urea cycle is incomplete. Arginine has to be converted into citrulline by arginine deiminase (EC: 3.5.3.6), forming a shunt (e.g. modes no. 22). An advantage shared by *S. saprophyticus *and *S. epidermidis *is that their native malate dehydrogenase (EC: 1.1.1.37) is present (e.g. modes no: 21, 22, 24 in *S. saprophyticus *and modes no. 16, 17, 18 in *S. epidermidis*), so that their citric acid cycle is more efficient and produces more ATP. These are only some strain-specific differences for illustration and exploiting the new software to show how the full scale metabolic models may be easily and quickly compared. In practice, after this initial analysis in most cases a more detailed study of individual sub-networks has to follow, often in combination with further genome re-annotation as well as experimental tests (e.g. [17]).

### Robustness analysis

Furthermore, our software provides a systematic test routine to get an estimate of the robustness of a metabolic network. The principle is to validate the flux preservation when a certain involved enzyme is knocked out from the system. Our program simulates this evolutionary or genetic event by removing enzymes one at a time and re-inspecting all generated flux modes. These survival modes after a single enzyme gene knock-out are used to predict the number of metabolites which can be still produced in the restricted system. Consequently all the enzymes are examined and an average metabolite production is calculated. By dividing the number through the maximum number of external metabolites which can be produced by the complete system, we achieve a percentage, referred to as the robustness score, which is an important feature of the system (see implementation). Table 1 illustrates robustness differences among these three systems. From the results (the third row), *S. saprophyticus *achieves the highest score (92.84), it is definitely the most robust organism among the three *Staphylococci *compared. This is potentially caused by its flexible living environments, not only *in vivo *as a pathogen but also outside any host. In contrast, *S. aureus *has strictly adapted to the host environment so that it requires more complex medium in order to acquire a recognizable growth rate.

## Discussion

In recent years, a number of stoichiometric network analyzers have been presented. The most prominent include "Metatool" [12], "FluxAnalyzer" [25], "Jarnac", a module for the systems biology workbench [26], "Gepasi" [27], "ScrumPy" [16] and "COPASI" [28]. Additionally the recent work by Urbanczik *et al*. introduces SNA, a stoichiometric analysis package using Mathematica [14]. These programs include sophisticated implementations of the algorithm for computation of the steady-state flux modes, or link to the efficient Metatool implementation. Some, for example "FluxAnalyzer", even provide a basic graphical view of the network but may require, like the SNA, a valid Matlab license to run.

With YANAsquare we introduce four new components to the previously published software, namely (i) an integrated EMA algorithm implemented in Java, (ii) a browser module for direct access to and download of pathways from KEGG, (iii) visualization and layout algorithms for metabolic networks and (iv) a robustness analysis algorithm and apply them all here to different example networks.

YANAsquare is a standalone open-source software and a 100% platform-independent modeling suite for steady-state analyses of metabolic networks. Besides the obligatory computation of the convex flux cone a variety of additional modules and algorithms are included. They tackle some of the most prominent challenges and questions in the analysis of large metabolic networks, such as dealing with the combinatorial explosion of elementary flux modes by application of different dissection strategies [15,29] or the newly implemented method to analyze network robustness. It directly integrates a graphical visualization of the network using field-tested layout algorithms and powerfully combines this approach with the possibility to query the KEGG database directly from the application.

The querying of pathway data is offered in two options, screening a local database or by performing remote calls to the KEGG server via the Internet. It includes a smart editor for reworking of the results as well as a database driven approach to abbreviate the often complicated and non-standardized compound names found in KEGG.

Despite its straightforwardness it has to be stressed that importing pathways from KEGG has to be done with some caution. Even though the ressource is a valuable tool and certainly eases the construction of metabolic networks as shown in our examples, networks should always be double-checked manually. The data offered in KEGG is not always reliable and may contain pathway or stoichiometric inconsistencies on which YANA performs only some basic checks. Additionally thorough literature research is mandatory to find out about variant pathways in the organism under study as the KEGG pathways are quite generic. Nevertheless it should be noted that the KGB module was never intended to be a replacement for a well-crafted manually set up metabolic network but to ease and guide the process of network reconstruction by quickly providing the user a sound data basis to work on. Besides the points mentioned the necessary manual reworking also includes the careful setting of the internal/external status of metabolites and the addition of any needed transport processes (see for example TransportDB, [30,31]) which are not part of KEGG at the time. Transport processes are easily incorporated into YANAsquare using reactions such as "Transporter1 : substrate_*ext *_→ substrate_*int*_" to indicate that transporter1 transports the substrate from outside to inside. Compartimentalization can also be studied by YANAsquare. Different compartments are easily incorporated and defined as different subnetworks in the software. Each subnetwork contains only those enzymatic reactions actually taking place in this compartment. Transport reactions between compartments (e.g. mitchondrion ↔ cytoplasm) are defined analogous to above.

The visualization routine allows easy rendering and drawing even of genome-scale networks, including standard editing possibilities like grid-alignment and zooming into interesting parts of the map. The force-directed lay-out was further improved to avoid overlaps in drawings and sketches.

The implemented robustness routine allows to rapidly investigate the effect of enzyme disruptions in terms of network stability (by drug effects or gene impairment). The question arises how significant the found differences are in robustness for the compared three genome-scale networks. A statistical test is difficult: The resulting elementary mode matrix and the resulting found differences are strictly deterministic. Instead one would like to investigate stochastic variation of the input stochiometric matrix. As the landscape for the obtained number of modes is very rugged, aleatoric changes in the matrix require an extreme high number of trials to get sufficient observations for statistical significance. Instead we compare here biological meaningful optimized stochiometric input matrices (decision of compounds and their status as internal or external according to biochemistry). This does not leave much space for variation here (e.g. change of one metabolite from internal to external is in most cases not biochemically justified). Thus we believe that the found differences correspond to realistic differences in robustness. However, in an applied experimental study we would in any case recommend to complement the prediction data from the routine with some genetic data (e.g. how many knockouts are tolerated in one strain compared to others).

## Conclusion

For each specific question in the field of metabolic modeling different implementations and solutions are feasible and every algorithm and software proposed so far has its own focus [12,14,16,25-27]. The present application develops important basic analysis algorithm further to efficiently tackle even large networks. As a modular open-source Java application it is freely available for all academic users and can be further extended or modified according to the needs of the individual users (the source code is contained within the download package).

We applied YANAsquare to model the complex signaling network in the murine phagosome, aiming at making predictions about compounds that activate phagosomal actin nucleation or fail to do so. Even though the network model shown here is a strong abstraction from the real signaling pathways in the living cell and the phagosome, most phospholipids indeed performed in experimental tests as activating or inhibiting actin nucleation, as predicted by our software in the simplified model.

*S. aureus *genome-scale analyses have been recently described [24,32]. We show in our examples that YANAsquare does not only allow to rapidly set-up the *S. aureus *primary metabolism, but that it is also suited for comparison of different *Staphylococci *species. This is by no means a substitute for detailed studies such as the two cited above but used here as an illustration example to stress the advantages of our software for rapid and efficient network setup and analysis where we compiled several clear differences found. In summary, the new capabilities of YANAsquare allow rapid setup, comparison and modelling of complex genome-sized networks including robustness tests, modifications and enhanced visualization. Future versions might include additional ways of compartmentalization, more advanced layout algorithms specifically suited for bipartite graphs and direct editing through the graph interface.

## Availability and requirements

• Project name: YANAsquare

• Project homepage: . After acceptance this will be the access for the reader to software and supplementary material. If published, the software application/tool will be readily available to any scientist wishing to use it for non-commercial purposes, without restrictions such as the need for a material transfer agreement.

• Operating Systems: Windows, Linux, General Unix, Macintosh

• Programming Language: Java

• Other Requirements: Java Runtime Environment 1.5 or higher

• License: GNU GPL

## Abbreviations

EMA, Elementary Mode Analysis; FBA, Flux Balance Analysis; KEGG, Kyoto Encyclopedia of Genes and Genomes; KGB, KEGG Browser; SOAP, Simple Object Access Protocol; TCA, Tricarboxylic Acid Cycle; WSDL, Web Services Description Language

## Authors' contributions

RS programmed and tested YANAsquare and its visualization routines and drafted the manuscript. CL implemented and tested the KEGG browser (KGB) module. CK provided the Java implementation of the elementary mode analysis algorithm. MK and SK designed and carried out experiments on the phagosomal phospholipids and verified our *in-silico *predictions. MH provided expert advice on *Staphylococci *and analysis of their metabolism. GG guided the study of the phagosomal phospholipids and provided his expertise on phospholipid networks for this work. SS developed algorithms for elementary mode analysis and aided CK in the efficient implementation of the algorithm in Java. TD advised, organized and guided the present study and drafted the manuscript. All authors read and approved the final manuscript.

## Supplementary Material

Additional file 1 Network definitions and results. Network definitions and results from the steady-state analyses used in this study. Contains the network definitions of the extended (Tab. 4–6) and the reduced (Tab. 1–3) murine phagosome system, as well as of the *S. saprophyticus *(Tab. 7–9), *S. aureus *COL/N315 (Tab. 10–12) and *S. epidermidis *(Tab. 13–15) species. To keep the tables concise only the Elementary Modes referenced in the text are included for the bacteria systems. The complete set of EMs is available from the authors on request. Click here for file
