# Supplementary material for: Integrated network reconstruction, visualization and analysis using YANAsquare
Source: BMC Bioinformatics. 2007 Aug 28;8:313. doi: 10.1186/1471-2105-8-313 (PMC2020486; doi:10.1186/1471-2105-8-313)
Supplement: Additional file 1 — Network definitions and results. Network definitions and results from the steady-state analyses used in this study. Contains the network definitions of the extended (Tab. 4–6) and the reduced (Tab. 1–3) murine phagosome system, as well as of the S. saprophyticus (Tab. 7–9), S. aureus COL/N315 (Tab. 10–12) and S. epidermidis (Tab. 13–15) species. To keep the tables concise only the Elementary Modes referenced in the text are included for the bacteria systems. The complete set of EMs is available from the authors on request. [file 1471-2105-8-313-S1.pdf]

Table 1: Reduced phagosome network - metabolites

Name	internal?	Connectivity
AA	false	1
Ac-sph	true	2
Actin	false	2
Actinpoly	false	2
ADP	true	7
ATP	true	7
CDP-cholin	false	2
CDP-DAG	true	2
cholin	true	3
cholinP	true	4
CMP	false	2
CTP	false	2
DAG	true	7
DAG-3P	false	1
ERM	true	2
ERM-PIP2	false	1
Ezrin	false	1
fatac	false	3
IcP	false	1
inositol	false	1
inositolTP	false	1
monoAGcholinP	true	2
P	false	2
PA	false	3
PC	false	4
PI	false	3
PIP	true	2
PIP2	true	3
PPi	false	2
S-1-P	false	1
sph	false	2
Sphmy	false	1
TAG	false	1
TM	false	1

Table 2: Reduced phagosome network - reactions

Enzyme name	reversible?	Reaction equation
Actindepoly	false	Actinpoly = 2 Actin
Actinnucl	false	ATP + 2 Actin = ADP + Actinpoly + P
CDPinotra	true	CDP-DAG + inositol = CMP + PI
CDPsynth	false	CTP + PA = CDP-DAG + PPi
ceramidse	true	Ac-sph = fatac + sph
Cholinkin	true	ATP + cholin = ADP + cholinP
CholinPtf	true	CTP + cholinP = CDP-cholin + PPi
DAGcholT	true	CMP + PC = CDP-cholin + DAG
DAGkin	true	ATP + DAG = ADP + DAG-3P
ERMfform	true	ERM + PIP2 = ERM-PIP2
ERMform	true	Ezrin + TM = ERM
Lipase	true	TAG = DAG + fatac
monoAGcholinPCtf	true	ADP + monoAGcholinP = ATP + cholin + fatac
PAP	true	PA = DAG + P
PI45diase	false	PIP2 = DAG + inositolTP
PI4kin	true	ATP + PI = ADP + PIP
PI4P5kin	true	ATP + PIP = ADP + PIP2
PIdiase	false	PI = DAG + IcP
PLA2	true	PC = AA + monoAGcholinP
PLC	true	PC = DAG + cholinP
PLD	true	PC = PA + cholin
Sphmydias	true	Sphmy = Ac-sph + cholinP
SPK	true	ATP + sph = ADP + S-1-P

Table 3: Reduced phagosome network - Elementary Modes[1]

#	Flux sum	Rev.?	Pathlength	Reactions	Net reaction
1	4	true	4	(-1 monoAGcholinPCtf) (-1 PLA2) (1 PLD) (-1 SPK)	AA + S-1-P + fatac = PA + sph
2	2	true	2	(1 Lipase) (-1 PAP)	P + TAG = PA + fatac
3	6	true	5	(-1 ERMfform) (-1 ERMform) (-1 PI4kin) (-1 PI4P5kin) (2 SPK)	ERM-PIP2 + 2 sph = Ezrin + PI + 2 S-1-P + TM
4	10	true	7	(-1 ERMfform) (-1 ERMform) (-2 monoAGcholinPCtf) (-1 PI4kin) (-1 PI4P5kin) (-2 PLA2) (2 PLD)	2 AA + ERM-PIP2 + 2 fatac = Ezrin + 2 PA + PI + TM
5	3	true	3	(-1 DAGkin) (-1 PAP) (1 SPK)	DAG-3P + P + sph = PA + S-1-P
6	5	true	5	(1 DAGkin) (1 monoAGcholinPCtf) (1 PAP) (1 PLA2) (-1 PLD)	2 PA = AA + DAG-3P + P + fatac
7	3	true	3	(-1 DAGkin) (-1 Lipase) (1 SPK)	DAG-3P + fatac + sph = S-1-P + TAG
8	5	true	5	(1 DAGkin) (1 Lipase) (1 monoAGcholinPCtf) (1 PLA2) (-1 PLD)	PA + TAG = AA + DAG-3P + 2 fatac
9	8	true	6	(-2 DAGkin) (1 ERMfform) (1 ERMform) (-2 PAP) (1 PI4kin) (1 PI4P5kin)	2 DAG-3P + Ezrin + 2 P + PI + TM = ERM-PIP2 + 2 PA
10	8	true	6	(-2 DAGkin) (1 ERMfform) (1 ERMform) (-2 Lipase) (1 PI4kin) (1 PI4P5kin)	2 DAG-3P + Ezrin + PI + TM + 2 fatac = ERM-PIP2 + 2 TAG
11	2	true	2	(1 DAGcholT) (-1 PAP)	CMP + P + PC = CDP-cholin + PA
12	2	true	2	(1 DAGcholT) (-1 Lipase)	CMP + PC + fatac = CDP-cholin + TAG
13	3	true	3	(-1 DAGcholT) (-1 DAGkin) (1 SPK)	CDP-cholin + DAG-3P + sph = CMP + PC + S-1-P
14	5	true	5	(1 DAGcholT) (1 DAGkin) (1 monoAGcholinPCtf) (1 PLA2) (-1 PLD)	CMP + PA + PC = AA + CDP-cholin + DAG-3P + fatac
15	8	true	6	(-2 DAGcholT) (-2 DAGkin) (1 ERMfform) (1 ERMform) (1 PI4kin) (1 PI4P5kin)	2 CDP-cholin + 2 DAG-3P + Ezrin + PI + TM = 2 CMP + ERM-PIP2 + 2 PC
16	3	true	3	(-1 CholinPtf) (1 PAP) (-1 PLC)	CDP-cholin + PA + PPi = CTP + P + PC
17	3	true	3	(-1 CholinPtf) (1 Lipase) (-1 PLC)	CDP-cholin + PPi + TAG = CTP + PC + fatac
18	4	true	4	(-1 CholinPtf) (-1 DAGkin) (-1 PLC) (1 SPK)	CDP-cholin + DAG-3P + PPi + sph = CTP + PC + S-1-P
19	6	true	6	(-1 CholinPtf) (-1 DAGkin) (-1 monoAGcholinPCtf) (-1 PLA2) (-1 PLC) (1 PLD)	AA + CDP-cholin + DAG-3P + PPi + fatac = CTP + PA + PC
20	10	true	7	(2 CholinPtf) (2 DAGkin) (-1 ERMfform) (-1 ERMform) (-1 PI4kin) (-1 PI4P5kin) (2 PLC)	2 CTP + ERM-PIP2 + 2 PC = 2 CDP-cholin + 2 DAG-3P + Ezrin + PI + 2 PPi + TM
21	3	true	3	(-1 CholinPtf) (1 DAGcholT) (-1 PLC)	CMP + PPi = CTP
22	5	true	5	(-1 Cholinkin) (-1 PAP) (1 PLC) (-1 PLD) (1 SPK)	P + sph = S-1-P
23	5	true	5	(-1 Cholinkin) (-1 monoAGcholinPCtf) (-1 PAP) (-1 PLA2) (1 PLC)	AA + P + fatac = PA
24	5	true	5	(-1 Cholinkin) (-1 Lipase) (1 PLC) (-1 PLD) (1 SPK)	PA + fatac + sph = S-1-P + TAG

25	5	true	5	(-1 Cholinkin) (-1 Lipase) (-1 monoAGcholinPCtf) (-1 PLA2) (1 PLC)	AA + 2 fatac = TAG
26	12	true	8	(2 Cholinkin) (-1 ERMfform) (-1 ERMform) (2 PAP) (-1 PI4kin) (-1 PI4P5kin) (-2 PLC) (2 PLD)	ERM-PIP2 = Ezrin + 2 P + PI + TM
27	12	true	8	(2 Cholinkin) (-1 ERMfform) (-1 ERMform) (2 Lipase) (-1 PI4kin) (-1 PI4P5kin) (-2 PLC) (2 PLD)	ERM-PIP2 + 2 TAG = Ezrin + 2 PA + PI + TM + 2 fatac
28	4	true	4	(-1 Cholinkin) (1 DAGkin) (1 PLC) (-1 PLD)	PA = DAG-3P
29	6	true	6	(-1 Cholinkin) (1 DAGkin) (-1 monoAGcholinPCtf) (-1 PLA2) (1 PLC) (-1 SPK)	AA + S-1-P + fatac = DAG-3P + sph
30	14	true	9	(-2 Cholinkin) (2 DAGkin) (-1 ERMfform) (-1 ERMform) (-2 monoAGcholinPCtf) (-1 PI4kin) (-1 PI4P5kin) (-2 PLA2) (2 PLC)	2 AA + ERM-PIP2 + 2 fatac = 2 DAG-3P + Ezrin + PI + TM
31	5	true	5	(-1 Cholinkin) (-1 DAGcholT) (1 PLC) (-1 PLD) (1 SPK)	CDP-cholin + PA + sph = CMP + PC + S-1-P
32	5	true	5	(-1 Cholinkin) (-1 DAGcholT) (-1 monoAGcholinPCtf) (-1 PLA2) (1 PLC)	AA + CDP-cholin + fatac = CMP + PC
33	12	true	8	(2 Cholinkin) (2 DAGcholT) (-1 ERMfform) (-1 ERMform) (-1 PI4kin) (-1 PI4P5kin) (-2 PLC) (2 PLD)	2 CMP + ERM-PIP2 + 2 PC = 2 CDP-cholin + Ezrin + 2 PA + PI + TM
34	4	true	4	(-1 Cholinkin) (-1 CholinPtf) (-1 PLD) (1 SPK)	CDP-cholin + PA + PPi + sph = CTP + PC + S-1-P
35	4	true	4	(-1 Cholinkin) (-1 CholinPtf) (-1 monoAGcholinPCtf) (-1 PLA2)	AA + CDP-cholin + PPi + fatac = CTP + PC
36	10	true	7	(2 Cholinkin) (2 CholinPtf) (-1 ERMfform) (-1 ERMform) (-1 PI4kin) (-1 PI4P5kin) (2 PLD)	2 CTP + ERM-PIP2 + 2 PC = 2 CDP-cholin + Ezrin + 2 PA + PI + 2 PPi + TM
37	5	true	5	(1 Cholinkin) (1 CholinPtf) (-1 DAGkin) (-1 PAP) (1 PLD)	CTP + DAG-3P + P + PC = CDP-cholin + 2 PA + PPi
38	5	true	5	(1 Cholinkin) (1 CholinPtf) (-1 DAGkin) (-1 Lipase) (1 PLD)	CTP + DAG-3P + PC + fatac = CDP-cholin + PA + PPi + TAG
39	5	true	5	(1 Cholinkin) (1 CholinPtf) (-1 DAGcholT) (-1 DAGkin) (1 PLD)	CTP + DAG-3P = CMP + PA + PPi
40	4	true	4	(-1 ceramidse) (-1 PAP) (1 PLC) (-1 Sphmydias)	P + PC + fatac + sph = PA + Sphmy
41	4	true	4	(-1 ceramidse) (-1 Lipase) (1 PLC) (-1 Sphmydias)	PC + 2 fatac + sph = Sphmy + TAG
42	5	true	5	(-1 ceramidse) (1 DAGkin) (1 PLC) (-1 Sphmydias) (-1 SPK)	PC + S-1-P + fatac = DAG-3P + Sphmy
43	7	true	7	(-1 ceramidse) (1 DAGkin) (1 monoAGcholinPCtf) (1 PLA2) (1 PLC) (-1 PLD) (-1 Sphmydias)	PA + PC + sph = AA + DAG-3P + Sphmy
44	12	true	8	(2 ceramidse) (-2 DAGkin) (1 ERMfform) (1 ERMform) (1 PI4kin) (1 PI4P5kin) (-2 PLC) (2 Sphmydias)	2 DAG-3P + Ezrin + PI + 2 Sphmy + TM = ERM-PIP2 + 2 PC + 2 fatac + 2 sph
45	4	true	4	(-1 ceramidse) (-1 DAGcholT) (1 PLC) (-1 Sphmydias)	CDP-cholin + fatac + sph = CMP + Sphmy
46	3	true	3	(1 ceramidse) (1 CholinPtf) (1 Sphmydias)	CTP + Sphmy = CDP-cholin + PPi + fatac + sph
47	5	true	5	(-1 ceramidse) (1 Cholinkin) (1 PLD) (-1 Sphmydias) (-1 SPK)	PC + S-1-P + fatac = PA + Sphmy

48	5	true	5	(-1 ceramidse) (1 Cholinkin) (1 monoAGcholinPCtf) (1 PLA2) (-1 Sphmydias)	PC + sph = AA + Sphmy
49	12	true	8	(2 ceramidse) (-2 Cholinkin) (1 ERMfform) (1 ERMform) (1 PI4kin) (1 PI4P5kin) (-2 PLD) (2 Sphmydias)	Ezrin + 2 PA + PI + 2 Sphmy + TM = ERM-PIP2 + 2 PC + 2 fatac + 2 sph
50	6	true	6	(1 ceramidse) (-1 Cholinkin) (1 DAGkin) (1 PAP) (-1 PLD) (1 Sphmydias)	2 PA + Sphmy = DAG-3P + P + PC + fatac + sph
51	6	true	6	(1 ceramidse) (-1 Cholinkin) (1 DAGkin) (1 Lipase) (-1 PLD) (1 Sphmydias)	PA + Sphmy + TAG = DAG-3P + PC + 2 fatac + sph
52	6	true	6	(1 ceramidse) (-1 Cholinkin) (1 DAGcholT) (1 DAGkin) (-1 PLD) (1 Sphmydias)	CMP + PA + Sphmy = CDP-cholin + DAG-3P + fatac + sph
53	2	false	2	(-1 PAP) (1 PIdiase)	P + PI = IcP + PA
54	2	false	2	(-1 Lipase) (1 PIdiase)	PI + fatac = IcP + TAG
55	3	false	3	(1 DAGkin) (1 PIdiase) (-1 SPK)	PI + S-1-P = DAG-3P + IcP + sph
56	5	false	5	(1 DAGkin) (1 monoAGcholinPCtf) (1 PIdiase) (1 PLA2) (-1 PLD)	PA + PI = AA + DAG-3P + IcP + fatac
57	8	false	6	(2 DAGkin) (-1 ERMfform) (-1 ERMform) (-1 PI4kin) (-1 PI4P5kin) (2 PIdiase)	ERM-PIP2 + PI = 2 DAG-3P + Ezrin + 2 IcP + TM
58	2	false	2	(-1 DAGcholT) (1 PIdiase)	CDP-cholin + PI = CMP + IcP + PC
59	3	false	3	(-1 CholinPtf) (1 PIdiase) (-1 PLC)	CDP-cholin + PI + PPi = CTP + IcP + PC
60	5	false	5	(1 Cholinkin) (1 PIdiase) (-1 PLC) (1 PLD) (-1 SPK)	PI + S-1-P = IcP + PA + sph
61	5	false	5	(1 Cholinkin) (1 monoAGcholinPCtf) (1 PIdiase) (1 PLA2) (-1 PLC)	PI = AA + IcP + fatac
62	12	false	8	(2 Cholinkin) (-1 ERMfform) (-1 ERMform) (-1 PI4kin) (-1 PI4P5kin) (2 PIdiase) (-2 PLC) (2 PLD)	ERM-PIP2 + PI = Ezrin + 2 IcP + 2 PA + TM
63	5	false	5	(-1 Cholinkin) (-1 CholinPtf) (1 DAGkin) (1 PIdiase) (-1 PLD)	CDP-cholin + PA + PI + PPi = CTP + DAG-3P + IcP + PC
64	4	false	4	(1 ceramidse) (1 PIdiase) (-1 PLC) (1 Sphmydias)	PI + Sphmy = IcP + PC + fatac + sph
65	6	false	6	(1 ceramidse) (-1 Cholinkin) (1 DAGkin) (1 PIdiase) (-1 PLD) (1 Sphmydias)	PA + PI + Sphmy = DAG-3P + IcP + PC + fatac + sph
66	6	false	5	(-1 PAP) (1 PI45diase) (1 PI4kin) (1 PI4P5kin) (-2 SPK)	P + PI + 2 S-1-P = PA + inositolTP + 2 sph
67	10	false	7	(2 monoAGcholinPCtf) (-1 PAP) (1 PI45diase) (1 PI4kin) (1 PI4P5kin) (2 PLA2) (-2 PLD)	P + PA + PI = 2 AA + 2 fatac + inositolTP
68	6	false	5	(-1 Lipase) (1 PI45diase) (1 PI4kin) (1 PI4P5kin) (-2 SPK)	PI + 2 S-1-P + fatac = TAG + inositolTP + 2 sph
69	10	false	7	(-1 Lipase) (2 monoAGcholinPCtf) (1 PI45diase) (1 PI4kin) (1 PI4P5kin) (2 PLA2) (-2 PLD)	2 PA + PI = 2 AA + TAG + fatac + inositolTP
70	4	false	4	(-1 ERMfform) (-1 ERMform) (-1 PAP) (1 PI45diase)	ERM-PIP2 + P = Ezrin + PA + TM + inositolTP
71	4	false	4	(-1 ERMfform) (-1 ERMform) (-1 Lipase) (1 PI45diase)	ERM-PIP2 + fatac = Ezrin + TAG + TM + inositolTP

72	7	false	5	(1 DAGkin) (1 PI45diase) (1 PI4kin) (1 PI4P5kin) (-3 SPK)	PI + 3 S-1-P = DAG-3P + inositolTP + 3 sph
73	8	false	5	(-2 DAGkin) (-3 PAP) (1 PI45diase) (1 PI4kin) (1 PI4P5kin)	2 DAG-3P + 3 P + PI = 3 PA + inositolTP
74	13	false	7	(1 DAGkin) (3 monoAGcholinPCtf) (1 PI45diase) (1 PI4kin) (1 PI4P5kin) (3 PLA2) (-3 PLD)	3 PA + PI = 3 AA + DAG-3P + 3 fatac + inositolTP
75	8	false	5	(-2 DAGkin) (-3 Lipase) (1 PI45diase) (1 PI4kin) (1 PI4P5kin)	2 DAG-3P + PI + 3 fatac = 3 TAG + inositolTP
76	5	false	5	(1 DAGkin) (-1 ERMfform) (-1 ERMform) (1 PI45diase) (-1 SPK)	ERM-PIP2 + S-1-P = DAG-3P + Ezrin + TM + inositolTP + sph
77	12	false	6	(2 DAGkin) (-3 ERMfform) (-3 ERMform) (2 PI45diase) (-1 PI4kin) (-1 PI4P5kin)	3 ERM-PIP2 = 2 DAG-3P + 3 Ezrin + PI + 3 TM + 2 inositolTP
78	7	false	7	(1 DAGkin) (-1 ERMfform) (-1 ERMform) (1 monoAGcholinPCtf) (1 PI45diase) (1 PLA2) (-1 PLD)	ERM-PIP2 + PA = AA + DAG-3P + Ezrin + TM + fatac + inositolTP
79	6	false	5	(-1 DAGcholT) (1 PI45diase) (1 PI4kin) (1 PI4P5kin) (-2 SPK)	CDP-cholin + PI + 2 S-1-P = CMP + PC + inositolTP + 2 sph
80	10	false	7	(-1 DAGcholT) (2 monoAGcholinPCtf) (1 PI45diase) (1 PI4kin) (1 PI4P5kin) (2 PLA2) (-2 PLD)	CDP-cholin + 2 PA + PI = 2 AA + CMP + PC + 2 fatac + inositolTP
81	4	false	4	(-1 DAGcholT) (-1 ERMfform) (-1 ERMform) (1 PI45diase)	CDP-cholin + ERM-PIP2 = CMP + Ezrin + PC + TM + inositolTP
82	8	false	5	(-3 DAGcholT) (-2 DAGkin) (1 PI45diase) (1 PI4kin) (1 PI4P5kin)	3 CDP-cholin + 2 DAG-3P + PI = 3 CMP + 3 PC + inositolTP
83	7	false	6	(-1 CholinPtf) (1 PI45diase) (1 PI4kin) (1 PI4P5kin) (-1 PLC) (-2 SPK)	CDP-cholin + PI + PPi + 2 S-1-P = CTP + PC + inositolTP + 2 sph
84	11	false	8	(-1 CholinPtf) (2 monoAGcholinPCtf) (1 PI45diase) (1 PI4kin) (1 PI4P5kin) (2 PLA2) (-1 PLC) (-2 PLD)	CDP-cholin + 2 PA + PI + PPi = 2 AA + CTP + PC + 2 fatac + inositolTP
85	5	false	5	(-1 CholinPtf) (-1 ERMfform) (-1 ERMform) (1 PI45diase) (-1 PLC)	CDP-cholin + ERM-PIP2 + PPi = CTP + Ezrin + PC + TM + inositolTP
86	11	false	6	(-3 CholinPtf) (-2 DAGkin) (1 PI45diase) (1 PI4kin) (1 PI4P5kin) (-3 PLC)	3 CDP-cholin + 2 DAG-3P + PI + 3 PPi = 3 CTP + 3 PC + inositolTP
87	9	false	7	(1 Cholinkin) (1 PI45diase) (1 PI4kin) (1 PI4P5kin) (-1 PLC) (1 PLD) (-3 SPK)	PI + 3 S-1-P = PA + inositolTP + 3 sph
88	12	false	7	(-2 Cholinkin) (-3 PAP) (1 PI45diase) (1 PI4kin) (1 PI4P5kin) (2 PLC) (-2 PLD)	3 P + PI = PA + inositolTP
89	9	false	8	(1 Cholinkin) (1 monoAGcholinPCtf) (1 PI45diase) (1 PI4kin) (1 PI4P5kin) (1 PLA2) (-1 PLC) (-2 SPK)	PI + 2 S-1-P = AA + fatac + inositolTP + 2 sph
90	13	false	8	(1 Cholinkin) (3 monoAGcholinPCtf) (1 PI45diase) (1 PI4kin) (1 PI4P5kin) (3 PLA2) (-1 PLC) (-2 PLD)	2 PA + PI = 3 AA + 3 fatac + inositolTP
91	12	false	7	(-2 Cholinkin) (-3 Lipase) (1 PI45diase) (1 PI4kin) (1 PI4P5kin) (2 PLC) (-2 PLD)	2 PA + PI + 3 fatac = 3 TAG + inositolTP

92	7	false	7	(1 Cholinkin) (-1 ERMfform) (-1 ERMform) (1 PI45diase) (-1 PLC) (1 PLD) (-1 SPK)	ERM-PIP2 + S-1-P = Ezrin + PA + TM + inositolTP + sph
93	16	false	8	(2 Cholinkin) (-3 ERMfform) (-3 ERMform) (2 PI45diase) (-1 PI4kin) (-1 PI4P5kin) (-2 PLC) (2 PLD)	3 ERM-PIP2 = 3 Ezrin + 2 PA + PI + 3 TM + 2 inositolTP
94	7	false	7	(1 Cholinkin) (-1 ERMfform) (-1 ERMform) (1 monoAGcholinPCtf) (1 PI45diase) (1 PLA2) (-1 PLC)	ERM-PIP2 = AA + Ezrin + TM + fatac + inositolTP
95	17	false	8	(3 Cholinkin) (-2 DAGkin) (3 monoAGcholinPCtf) (1 PI45diase) (1 PI4kin) (1 PI4P5kin) (3 PLA2) (-3 PLC)	2 DAG-3P + PI = 3 AA + 3 fatac + inositolTP
96	12	false	7	(-2 Cholinkin) (-3 DAGcholT) (1 PI45diase) (1 PI4kin) (1 PI4P5kin) (2 PLC) (-2 PLD)	3 CDP-cholin + 2 PA + PI = 3 CMP + 3 PC + inositolTP
97	11	false	7	(-2 Cholinkin) (-3 CholinPtf) (1 PI45diase) (1 PI4kin) (1 PI4P5kin) (-1 PLC) (-2 PLD)	3 CDP-cholin + 2 PA + PI + 3 PPi = 3 CTP + 3 PC + inositolTP
98	10	false	7	(-2 Cholinkin) (-2 CholinPtf) (-1 PAP) (1 PI45diase) (1 PI4kin) (1 PI4P5kin) (-2 PLD)	2 CDP-cholin + P + PA + PI + 2 PPi = 2 CTP + 2 PC + inositolTP
99	10	false	7	(-2 Cholinkin) (-2 CholinPtf) (-1 Lipase) (1 PI45diase) (1 PI4kin) (1 PI4P5kin) (-2 PLD)	2 CDP-cholin + 2 PA + PI + 2 PPi + fatac = 2 CTP + 2 PC + TAG + inositolTP
100	13	false	7	(-3 Cholinkin) (-3 CholinPtf) (1 DAGkin) (1 PI45diase) (1 PI4kin) (1 PI4P5kin) (-3 PLD)	3 CDP-cholin + 3 PA + PI + 3 PPi = 3 CTP + DAG-3P + 3 PC + inositolTP
101	7	false	7	(-1 Cholinkin) (-1 CholinPtf) (1 DAGkin) (-1 ERMfform) (-1 ERMform) (1 PI45diase) (-1 PLD)	CDP-cholin + ERM-PIP2 + PA + PPi = CTP + DAG-3P + Ezrin + PC + TM + inositolTP
102	10	false	7	(-2 Cholinkin) (-2 CholinPtf) (-1 DAGcholT) (1 PI45diase) (1 PI4kin) (1 PI4P5kin) (-2 PLD)	3 CDP-cholin + 2 PA + PI + 2 PPi = CMP + 2 CTP + 3 PC + inositolTP
103	8	false	7	(1 ceramidse) (1 PI45diase) (1 PI4kin) (1 PI4P5kin) (-1 PLC) (1 Sphmydias) (-2 SPK)	PI + 2 S-1-P + Sphmy = PC + fatac + inositolTP + 3 sph
104	12	false	9	(1 ceramidse) (2 monoAGcholinPCtf) (1 PI45diase) (1 PI4kin) (1 PI4P5kin) (2 PLA2) (-1 PLC) (-2 PLD) (1 Sphmydias)	2 PA + PI + Sphmy = 2 AA + PC + 3 fatac + inositolTP + sph
105	6	false	6	(1 ceramidse) (-1 ERMfform) (-1 ERMform) (1 PI45diase) (-1 PLC) (1 Sphmydias)	ERM-PIP2 + Sphmy = Ezrin + PC + TM + fatac + inositolTP + sph
106	14	false	7	(3 ceramidse) (-2 DAGkin) (1 PI45diase) (1 PI4kin) (1 PI4P5kin) (-3 PLC) (3 Sphmydias)	2 DAG-3P + PI + 3 Sphmy = 3 PC + 3 fatac + inositolTP + 3 sph
107	14	false	8	(3 ceramidse) (-2 Cholinkin) (1 PI45diase) (1 PI4kin) (1 PI4P5kin) (-1 PLC) (-2 PLD) (3 Sphmydias)	2 PA + PI + 3 Sphmy = 3 PC + 3 fatac + inositolTP + 3 sph
108	12	false	8	(2 ceramidse) (-2 Cholinkin) (-1 PAP) (1 PI45diase) (1 PI4kin) (1 PI4P5kin) (-2 PLD) (2 Sphmydias)	P + PA + PI + 2 Sphmy = 2 PC + 2 fatac + inositolTP + 2 sph
109	12	false	8	(2 ceramidse) (-2 Cholinkin) (-1 Lipase) (1 PI45diase) (1 PI4kin) (1 PI4P5kin) (-2 PLD) (2 Sphmydias)	2 PA + PI + 2 Sphmy = 2 PC + TAG + fatac + inositolTP + 2 sph
110	16	false	8	(3 ceramidse) (-3 Cholinkin) (1 DAGkin) (1 PI45diase) (1 PI4kin) (1 PI4P5kin) (-3 PLD) (3 Sphmydias)	3 PA + PI + 3 Sphmy = DAG-3P + 3 PC + 3 fatac + inositolTP + 3 sph

111	8	false	8	(1 ceramidse) (-1 Cholinkin) (1 DAGkin) (-1 ERMfform) (-1 ERMform) (1 PI45diase) (-1 PLD) (1 Sphmydias)	ERM-PIP2 + PA + Sphmy = DAG-3P + Ezrin + PC + TM + fatac + inositolTP + sph
112	12	false	8	(2 ceramidse) (-2 Cholinkin) (-1 DAGcholT) (1 PI45diase) (1 PI4kin) (1 PI4P5kin) (-2 PLD) (2 Sphmydias)	CDP-cholin + 2 PA + PI + 2 Sphmy = CMP + 3 PC + 2 fatac + inositolTP + 2 sph
113	2	false	2	(1 CDPinotra) (1 CDPsynth)	CTP + PA + inositol = CMP + PI + PPi
114	2	false	2	(1 Actinnucl) (-1 SPK)	2 Actin + S-1-P = Actinpoly + P + sph
115	4	false	4	(1 Actinnucl) (1 monoAGcholinPCtf) (1 PLA2) (-1 PLD)	2 Actin + PA = AA + Actinpoly + P + fatac
116	6	false	5	(2 Actinnucl) (-1 ERMfform) (-1 ERMform) (-1 PI4kin) (-1 PI4P5kin)	4 Actin + ERM-PIP2 = 2 Actinpoly + Ezrin + 2 P + PI + TM
117	3	false	3	(1 Actinnucl) (-1 DAGkin) (-1 PAP)	2 Actin + DAG-3P = Actinpoly + PA
118	3	false	3	(1 Actinnucl) (-1 DAGkin) (-1 Lipase)	2 Actin + DAG-3P + fatac = Actinpoly + P + TAG
119	3	false	3	(1 Actinnucl) (-1 DAGcholT) (-1 DAGkin)	2 Actin + CDP-cholin + DAG-3P = Actinpoly + CMP + P + PC
120	4	false	4	(1 Actinnucl) (-1 CholinPtf) (-1 DAGkin) (-1 PLC)	2 Actin + CDP-cholin + DAG-3P + PPi = Actinpoly + CTP + P + PC
121	5	false	5	(1 Actinnucl) (-1 Cholinkin) (-1 PAP) (1 PLC) (-1 PLD)	2 Actin = Actinpoly
122	5	false	5	(1 Actinnucl) (-1 Cholinkin) (-1 Lipase) (1 PLC) (-1 PLD)	2 Actin + PA + fatac = Actinpoly + P + TAG
123	6	false	6	(1 Actinnucl) (1 Cholinkin) (-1 DAGkin) (1 monoAGcholinPCtf) (1 PLA2) (-1 PLC)	2 Actin + DAG-3P = AA + Actinpoly + P + fatac
124	5	false	5	(1 Actinnucl) (-1 Cholinkin) (-1 DAGcholT) (1 PLC) (-1 PLD)	2 Actin + CDP-cholin + PA = Actinpoly + CMP + P + PC
125	4	false	4	(1 Actinnucl) (-1 Cholinkin) (-1 CholinPtf) (-1 PLD)	2 Actin + CDP-cholin + PA + PPi = Actinpoly + CTP + P + PC
126	5	false	5	(1 Actinnucl) (1 ceramidse) (-1 DAGkin) (-1 PLC) (1 Sphmydias)	2 Actin + DAG-3P + Sphmy = Actinpoly + P + PC + fatac + sph
127	5	false	5	(1 Actinnucl) (1 ceramidse) (-1 Cholinkin) (-1 PLD) (1 Sphmydias)	2 Actin + PA + Sphmy = Actinpoly + P + PC + fatac + sph
128	1	false	1	(1 Actindepoly)	Actinpoly = 2 Actin

[1] *The actin nucleation modes are listed from mode 114 onwards. Only these phospholipids did also stimulate actin nucleation in experiments with phagosomes.*

Table 4: Extended phagosome network - metabolites

Name	internal?	Connectivity	Description
1-Acyl-sn-glycero-3-Pcholine	true	3	1-Acyl-sn-glycero-3-phosphocholine
1-Acyl-sn-glycero-3-PEtOHamine	true	2	1-Acyl-sn-glycero-3-phosphoethanolamine
1-Acyl-sn-glycerol3-P	false	2	1-Acyl-sn-glycerol 3-phosphate
12-Diacyl-sn-glycerol	true	4	1,2-Diacyl-sn-glycerol
2-Acyl-sn-glycero-3-Pcholine	false	1	2-Acyl-sn-glycero-3-phosphocholine
2-Acyl-sn-glycero-3-PEtOHamine	false	1	2-Acyl-sn-glycero-3-phosphoethanolamine
2-Acyl-sn-glycerol3-P	false	1	2-Acyl-sn-glycerol 3-phosphate
Ac	false	1	Acetate
Ac-CoA	false	1	Acetyl-CoA
Accholine	true	2	Acetylcholine
Acyl-CoA	false	4	Acyl-CoA
AcylDHAP	false	1	Acylglycerone phosphate
Adenosyl-L-homoCys	false	1	S-Adenosyl-L-homocysteine
Adenosyl-L-Met	false	1	S-Adenosyl-L-methionine
Cardiolipin	false	2	Cardiolipin
CDP-choline	false	2	CDP-choline
CDP-diacylglycerol	true	3	CDP-diacylglycerol
CDP-EtOHamine	false	1	CDP-ethanolamine
Choline	true	6	Choline
CholineP	true	3	Choline phosphate
CMP	false	3	CMP
CoA	false	5	CoA
CTP	false	3	CTP
D-Glucuronate	false	1	D-Glucuronate
DHAP	true	3	Glycerone phosphate
EtOHamine	true	4	Ethanolamine
EtOHamineP	true	3	Ethanolamine phosphate
FAD	false	1	FAD
FADH2	false	1	FADH2
Fattyacid	false	6	Fatty acid
G6P	false	1	D-Glucose 6-phosphate
Glycerol	false	1	Glycerol
inositol	true	5	myo-Inositol
inositol_TEP_1345	true	3	D-myo-Inositol 1,3,4,5-tetrakisphosphate
inositolBP_14	true	2	D-myo-Inositol 1,4-bisphosphate
inositolBP_34	true	2	D-myo-Inositol 3,4-bisphosphate
inositolHP	false	1	myo-Inositol hexakisphosphate

inositolP_1	true	2	Inositol 1-phosphate
inositolP_3	true	2	D-myo-Inositol 3-phosphate
inositolP_4	true	2	myo-Inositol 4-phosphate
inositolPP_13456	true	2	D-myo-Inositol 1,3,4,5,6-pentakisphosphate
inositolTEP_1456	true	2	D-myo-Inositol 1,4,5,6-tetrakisphosphate
inositolTP_134	true	2	D-myo-Inositol 1,3,4-trisphosphate
inositolTP_145	true	5	D-myo-Inositol 1,4,5-trisphosphate
L-Ser	false	2	L-Serine
NAD+	false	1	NAD+
NADH	false	1	NADH
Oxygen	false	1	Oxygen
PA	true	6	Phosphatidate
PC	true	5	Phosphatidylcholine
Phosphatidyl-N-methylEtOHamine	false	1	Phosphatidyl-N-methylethanolamine
PhosphatidylEtOHamine	true	5	Phosphatidylethanolamine
Phosphatidylglycerol	false	2	Phosphatidylglycerol
PI	false	3	1-Phosphatidyl-D-myo-inositol
PIP2_45	true	5	1-Phosphatidyl-D-myo-inositol 4,5-bisphosphate
PIP3_345	true	2	Phosphatidylinositol-3,4,5-trisphosphate
PIP_3	false	1	1-Phosphatidyl-1D-myo-inositol 3-phosphate
PIP_4	true	3	1-Phosphatidyl-1D-myo-inositol 4-phosphate
PSer	true	3	Phosphatidylserine
sn-glycero-3-Pcholine	false	2	sn-glycero-3-Phosphocholine
sn-glycero-3-PEtOHamine	false	2	sn-glycero-3-Phosphoethanolamine
sn-Glycerol3-P	true	4	sn-Glycerol 3-phosphate
Sterol	false	1	Sterol
Sterylester	false	1	Steryl ester

Table 5: Extended phagosome network - reactions

Enzyme name	rev.?	Reaction equation	Annotation
rn:R00840	true	inositolP_1 = G6P	1L-myo-Inositol-1-phosphate lyase (isomerizing) [5.5.1.4]
rn:R00842	false	NAD+ + sn-Glycerol3-P = DHAP + NADH	sn-Glycerol-3-phosphate:NAD+ 2-oxidoreductase [1.1.1.8 , 1.1.1.94]
rn:R00848	false	FAD + sn-Glycerol3-P = DHAP + FADH2	sn-Glycerol-3-phosphate:(acceptor) 2-oxidoreductase [1.1.99.5]
rn:R00851	false	Acyl-CoA + sn-Glycerol3-P = 1-Acyl-sn-glycerol3-P + CoA	acyl-CoA:sn-glycerol-3-phosphate 1-O-acyltransferase [2.3.1.15]
rn:R01013	true	Acyl-CoA + DHAP = AcylDHAP + CoA	Acyl-CoA:glycerone-phosphate O-acyltransferase [2.3.1.42]
rn:R01021	false	Choline = CholineP	ATP:choline phosphotransferase [2.7.1.32]
rn:R01023	false	Ac-CoA + Choline = Accholine + CoA	Acetyl-CoA:choline O-acetyltransferase [2.3.1.6]
rn:R01026	false	Accholine = Ac + Choline	Acetylcholine aectylhydrolase [3.1.1.7]
rn:R01184	false	Oxygen + inositol = D-Glucuronate	myo-Inositol:oxygen oxidoreductase [1.13.99.1]
rn:R01185	false	inositolP_1 = inositol	myo-Inositol 1-phosphate phosphahydrolase [3.1.3.25]
rn:R01186	false	inositolP_4 = inositol	myo-Inositol 4-phosphate phosphahydrolase [3.1.3.25]
rn:R01187	false	inositolP_3 = inositol	1D-myo-Inositol 3-phosphate phosphahydrolase [3.1.3.25]
rn:R01310	false	PC = Choline + PA	Phosphatidylcholine phosphatidohydrolase [3.1.4.4]
rn:R01315	false	PC = 1-Acyl-sn-glycero-3-Pcholine + Fattyacid	Phosphatidylcholine 2-acylhydrolase [3.1.1.4]
rn:R01321	false	12-Diacyl-sn-glycerol + CDP-choline = CMP + PC	CDPcholine:1,2-diacylglycerol cholinephosphotransferase [2.7.8.2]
rn:R01468	false	EtOHamine = EtOHamineP	ATP:ethanolamine O-phosphotransferase [2.7.1.82]
rn:R01799	false	CTP + PA = CDP-diacylglycerol	CTP:phosphatidate cytidyltransferase [2.7.7.41]
rn:R01802	false	CDP-diacylglycerol + inositol = CMP + PI	CDPdiacylglycerol:myo-inositol 3-phosphatidyltransferase [2.7.8.11]
rn:R01890	false	CTP + CholineP = CDP-choline	CTP:choline-phosphate cytidylyltransferase [2.7.7.15]
rn:R02030	false	CDP-diacylglycerol + Phosphatidylglycerol = CMP + Cardiolipin	[2.7.8.-]
rn:R02038	false	CTP + EtOHamineP = CDP-EtOHamine	CTP:ethanolamine-phosphate cytidylyltransferase [2.7.7.14]
rn:R02051	false	PhosphatidylEtOHamine = EtOHamine + PA	Phosphatidylethanolamine phosphatidohydrolase [3.1.4.4]
rn:R02053	false	PhosphatidylEtOHamine = 1-Acyl-sn-glycero-3-PEtOHamine + Fattyacid	Phosphatidylethanolamine 2-acylhydrolase [3.1.1.4]
rn:R02055	false	PSer = PhosphatidylEtOHamine	Phsophatidyl-L-serine carboxy-lyase [4.1.1.65]
rn:R02056	false	Adenosyl-L-Met + PhosphatidylEtOHamine = Adenosyl-L-homoCys + Phosphatidyl-N-methylEtOHamine	S-Adenosyl-L-methionine:phosphatidylethanolamine N-methyltransferase [2.1.1.17]
rn:R02114	false	PC + Sterol = 1-Acyl-sn-glycero-3-Pcholine + Sterylester	Phosphatidylcholine:sterol O-acyltransferase [2.3.1.43]
rn:R02239	false	PA = 12-Diacyl-sn-glycerol	1,2-Diacyl-sn-glycerol 3-phosphate phosphohydrolase [3.1.3.4]
rn:R02240	false	12-Diacyl-sn-glycerol = PA	ATP:1,2-diacylglycerol 3-phosphotransferase [2.7.1.107]
rn:R02241	false	CoA + PA = 1-Acyl-sn-glycerol3-P + Acyl-CoA	[2.3.1.51]

rn:R02746	false	1-Acyl-sn-glycero-3-Pcholine = Fattyacid + sn-glycero-3-Pcholine	1-Acyl-sn-glycero-3-phosphocholine acylhydrolase [3.1.1.5]
rn:R02747	false	2-Acyl-sn-glycero-3-Pcholine = Fattyacid + sn-glycero-3-Pcholine	2-Acyl-sn-glycero-3-phosphocholine acylhydrolase [3.1.1.5]
rn:R03361	false	PI = PIP_4	ATP:1-Phosphatidyl-1D-myo-inositol 4-phosphotransferase [2.7.1.67]
rn:R03362	false	PI = PIP_3	ATP:1-phosphatidyl-1D-myo-inositol 3-phosphotransferase [2.7.1.137]
rn:R03393	false	inositolBP_14 = inositolP_4	D-myo-Inositol-1,4-bisphosphate 1-phosphohydrolase [3.1.3.57]
rn:R03394	false	inositolTP_145 = inositolBP_14	D-myo-Inositol-1,4,5-trisphosphate 5-phosphohydrolase [3.1.3.56]
rn:R03416	false	1-Acyl-sn-glycero-3-PEtOHamine = Fattyacid + sn-glycero-3-PEtOHamine	1-Acyl-sn-glycero-3-phosphoethanolamine aldehydohydrolase [3.1.1.5]
rn:R03417	false	2-Acyl-sn-glycero-3-PEtOHamine = Fattyacid + sn-glycero-3-PEtOHamine	L-2-Lysophosphatidylethanolamine aldehydohydrolase [3.1.1.5]
rn:R03427	false	inositolTP_134 = inositolBP_34	D-myo-Inositol 1,3,4-trisphosphate 1-phosphohydrolase [3.1.3.57]
rn:R03430	false	inositol_TEP_1345 = inositolTP_134	D-myo-Inositol 1,3,4,5-tetrakisphosphate 5-phosphohydrolase [3.1.3.56]
rn:R03433	false	inositolTP_145 = inositol_TEP_1345	ATP:1D-myo-inositol-1,4,5-trisphosphate 3-phosphotransferase [2.7.1.127]
rn:R03434	false	inositol_TEP_1345 = inositolTP_145	D-myo-Inositol 1,3,4,5-tetrakisphosphate 3-phosphohydrolase [3.1.3.62]
rn:R03435	false	PIP2_45 = 12-Diacyl-sn-glycerol + inositolTP_145	1-Phosphatidyl-1D-myo-inositol-4,5-bisphosphate inositoltrisphosphohydrolase [3.1.4.11]
rn:R03469	false	PIP_4 = PIP2_45	ATP:1-phosphatidyl-1D-myo-inositol-4-phosphate 5-phosphotransferase [2.7.1.68]
rn:R04372	false	inositolBP_34 = inositolP_3	D-myo-Inositol-3,4-bisphosphate 4-phosphohydrolase [3.1.3.66]
rn:R04404	false	PIP2_45 = PIP_4	Phosphatidyl-myo-inositol-4,5-bisphosphate 4-phosphohydrolase [3.1.3.36]
rn:R04513	false	PIP3_345 = PIP2_45	Phosphatidylinositol-3,4,5-trisphosphate 3-phosphohydrolase [3.1.3.67]
rn:R04545	false	PIP2_45 = PIP3_345	ATP:1-phosphatidyl-1D-myo-inositol-4,5-bisphosphate 3-phosphotransferase [2.7.1.153]
rn:R05202	true	inositolHP = inositolPP_13456	unknown [2.7.1.-]
rn:R05800	false	inositolTP_145 = inositolTEP_1456	ATP:1D-myo-inositol-1,4,5-trisphosphate 6-phosphotransferase [2.7.1.151]
rn:R05801	false	inositolTEP_1456 = inositolPP_13456	ATP:1D-myo-inositol-1,4,5-trisphosphate 6-phosphotransferase [2.7.1.151]
rn:R06870	false	EtOHamineP = EtOHamine	Phosphoethanolamine phosphohydrolase [3.1.3.75]
rn:R06871	false	CholineP = Choline	Phosphocholine phosphohydrolase [3.1.3.75]

rn:R06872	true	Acyl-CoA + sn-Glycerol3-P = 2-Acyl-sn-glycerol3-P + CoA	Acyl-CoA:sn-glycerol-3-phosphate 2-O-acyltransferase []
rn:R07376	false	L-Ser + PhosphatidylEtOHamine = EtOHamine + PSer	[]
rn:R07377	false	L-Ser + PC = Choline + PSer	[]
rn:R07390	false	2 Phosphatidylglycerol = Cardiolipin + Glycerol	[2.7.8.-]

Table 6: Extended phagosome network - Elementary Modes

#	Flux sum	Rev.?	Pathlength	Reactions	Net reaction
1	1	false	1	(1 rn:R03417)	2-Acyl-sn-glycero-3-PEtOHamine = Fattyacid + sn-glycero-3-PEtOHamine
2	1	false	1	(1 rn:R07390)	2 Phosphatidylglycerol = Cardiolipin + Glycerol
3	3	false	3	(-1 rn:R00840) (1 rn:R01184) (1 rn:R01185)	G6P + Oxygen = D-Glucuronate
4	2	false	2	(1 rn:R00851) (-1 rn:R06872)	2-Acyl-sn-glycerol3-P = 1-Acyl-sn-glycerol3-P
5	2	false	2	(1 rn:R04513) (1 rn:R04545)	no net reaction
6	3	false	3	(1 rn:R00848) (1 rn:R01013) (-1 rn:R06872)	2-Acyl-sn-glycerol3-P + FAD = AcylDHAP + FADH2
7	3	false	3	(1 rn:R00842) (1 rn:R01013) (-1 rn:R06872)	2-Acyl-sn-glycerol3-P + NAD+ = AcylDHAP + NADH
8	2	false	2	(1 rn:R01468) (1 rn:R06870)	no net reaction
9	1	false	1	(1 rn:R03362)	PI = PIP_3
10	1	false	1	(1 rn:R02747)	2-Acyl-sn-glycero-3-Pcholine = Fattyacid + sn-glycero-3-Pcholine
11	2	false	2	(1 rn:R01021) (1 rn:R06871)	no net reaction
12	4	false	4	(1 rn:R01468) (1 rn:R02038) (1 rn:R02055) (1 rn:R07376)	CTP + L-Ser = CDP-EtOHamine
13	5	false	5	(1 rn:R01021) (1 rn:R01310) (1 rn:R01321) (1 rn:R01890) (1 rn:R02239)	CTP = CMP
14	2	false	2	(1 rn:R01023) (1 rn:R01026)	Ac-CoA = Ac + CoA
15	9	false	9	(1 rn:R01021) (1 rn:R01321) (1 rn:R01468) (1 rn:R01890) (1 rn:R02038) (1 rn:R02051) (1 rn:R02055) (1 rn:R02239) (1 rn:R07377)	2 CTP + L-Ser = CDP-EtOHamine + CMP
16	2	false	2	(1 rn:R03433) (1 rn:R03434)	no net reaction
17	2	false	2	(1 rn:R02239) (1 rn:R02240)	no net reaction
18	11	false	11	(1 rn:R01021) (1 rn:R01310) (1 rn:R01321) (1 rn:R01890) (1 rn:R02241) (1 rn:R03361) (1 rn:R03435) (1 rn:R03469) (-1 rn:R05202) (1 rn:R05800) (1 rn:R05801)	CTP + CoA + PI = 1-Acyl-sn-glycerol3-P + Acyl-CoA + CMP + inositolHP
19	12	false	12	(1 rn:R01021) (1 rn:R01310) (1 rn:R01321) (1 rn:R01799) (1 rn:R01890) (1 rn:R02030) (1 rn:R03361) (1 rn:R03435) (1 rn:R03469) (-1 rn:R05202) (1 rn:R05800) (1 rn:R05801)	2 CTP + PI + Phosphatidylglycerol = 2 CMP + Cardiolipin + inositolHP
20	14	false	14	(-1 rn:R00840) (1 rn:R01021) (1 rn:R01185) (1 rn:R01310) (1 rn:R01321) (1 rn:R01799) (1 rn:R01802) (1 rn:R01890) (1 rn:R03361) (1 rn:R03435) (1 rn:R03469) (-1 rn:R05202) (1 rn:R05800) (1 rn:R05801)	2 CTP + G6P = 2 CMP + inositolHP
21	14	false	14	(1 rn:R01021) (1 rn:R01184) (1 rn:R01187) (1 rn:R01310) (1 rn:R01321) (1 rn:R01890) (1 rn:R02241) (1 rn:R03361) (1 rn:R03427) (1 rn:R03430) (1 rn:R03433) (1 rn:R03435) (1 rn:R03469) (1 rn:R04372)	CTP + CoA + Oxygen + PI = 1-Acyl-sn-glycerol3-P + Acyl-CoA + CMP + D-Glucuronate

22	15	false	15	(1 rn:R01021) (1 rn:R01184) (1 rn:R01187) (1 rn:R01310) (1 rn:R01321) (1 rn:R01799) (1 rn:R01890) (1 rn:R02030) (1 rn:R03361) (1 rn:R03427) (1 rn:R03430) (1 rn:R03433) (1 rn:R03435) (1 rn:R03469) (1 rn:R04372)	2 CTP + Oxygen + PI + Phosphatidylglycerol = 2 CMP + Cardiolipin + D-Glucuronate
23	14	false	14	(1 rn:R01021) (1 rn:R01187) (1 rn:R01310) (1 rn:R01321) (1 rn:R01799) (1 rn:R01802) (1 rn:R01890) (1 rn:R03361) (1 rn:R03427) (1 rn:R03430) (1 rn:R03433) (1 rn:R03435) (1 rn:R03469) (1 rn:R04372)	2 CTP = 2 CMP
24	12	false	12	(1 rn:R01021) (1 rn:R01321) (1 rn:R01890) (1 rn:R02055) (1 rn:R02056) (1 rn:R03361) (1 rn:R03435) (1 rn:R03469) (-1 rn:R05202) (1 rn:R05800) (1 rn:R05801) (1 rn:R07377)	Adenosyl-L-Met + CTP + L-Ser + PI = Adenosyl-L-homoCys + CMP + Phosphatidyl-N-methylEtOHamine + inositolHP
25	15	false	15	(1 rn:R01021) (1 rn:R01184) (1 rn:R01187) (1 rn:R01321) (1 rn:R01890) (1 rn:R02055) (1 rn:R02056) (1 rn:R03361) (1 rn:R03427) (1 rn:R03430) (1 rn:R03433) (1 rn:R03435) (1 rn:R03469) (1 rn:R04372) (1 rn:R07377)	Adenosyl-L-Met + CTP + L-Ser + Oxygen + PI = Adenosyl-L-homoCys + CMP + D-Glucuronate + Phosphatidyl-N-methylEtOHamine
26	2	false	2	(1 rn:R03469) (1 rn:R04404)	no net reaction
27	15	false	15	(1 rn:R01021) (1 rn:R01321) (1 rn:R01468) (1 rn:R01890) (1 rn:R02038) (1 rn:R02051) (1 rn:R02055) (1 rn:R02241) (1 rn:R03361) (1 rn:R03435) (1 rn:R03469) (-1 rn:R05202) (1 rn:R05800) (1 rn:R05801) (1 rn:R07377)	2 CTP + CoA + L-Ser + PI = 1-Acyl-sn-glycerol3-P + Acyl-CoA + CDP-EtOHamine + CMP + inositolHP
28	16	false	16	(1 rn:R01021) (1 rn:R01321) (1 rn:R01468) (1 rn:R01799) (1 rn:R01890) (1 rn:R02030) (1 rn:R02038) (1 rn:R02051) (1 rn:R02055) (1 rn:R03361) (1 rn:R03435) (1 rn:R03469) (-1 rn:R05202) (1 rn:R05800) (1 rn:R05801) (1 rn:R07377)	3 CTP + L-Ser + PI + Phosphatidylglycerol = CDP-EtOHamine + 2 CMP + Cardiolipin + inositolHP
29	18	false	18	(-1 rn:R00840) (1 rn:R01021) (1 rn:R01185) (1 rn:R01321) (1 rn:R01468) (1 rn:R01799) (1 rn:R01802) (1 rn:R01890) (1 rn:R02038) (1 rn:R02051) (1 rn:R02055) (1 rn:R03361) (1 rn:R03435) (1 rn:R03469) (-1 rn:R05202) (1 rn:R05800) (1 rn:R05801) (1 rn:R07377)	3 CTP + G6P + L-Ser = CDP-EtOHamine + 2 CMP + inositolHP
30	18	false	18	(1 rn:R01021) (1 rn:R01184) (1 rn:R01187) (1 rn:R01321) (1 rn:R01468) (1 rn:R01890) (1 rn:R02038) (1 rn:R02051) (1 rn:R02055) (1 rn:R02241) (1 rn:R03361) (1 rn:R03427) (1 rn:R03430) (1 rn:R03433) (1 rn:R03435) (1 rn:R03469) (1 rn:R04372) (1 rn:R07377)	2 CTP + CoA + L-Ser + Oxygen + PI = 1-Acyl-sn-glycerol3-P + Acyl-CoA + CDP-EtOHamine + CMP + D-Glucuronate
31	19	false	19	(1 rn:R01021) (1 rn:R01184) (1 rn:R01187) (1 rn:R01321) (1 rn:R01468) (1 rn:R01799) (1 rn:R01890) (1 rn:R02030) (1 rn:R02038) (1 rn:R02051) (1 rn:R02055) (1 rn:R03361) (1 rn:R03427) (1 rn:R03430) (1 rn:R03433) (1 rn:R03435) (1 rn:R03469) (1 rn:R04372) (1 rn:R07377)	3 CTP + L-Ser + Oxygen + PI + Phosphatidylglycerol = CDP-EtOHamine + 2 CMP + Cardiolipin + D-Glucuronate

32	18	false	18	(1 rn:R01021) (1 rn:R01187) (1 rn:R01321) (1 rn:R01468) (1 rn:R01799) (1 rn:R01802) (1 rn:R01890) (1 rn:R02038) (1 rn:R02051) (1 rn:R02055) (1 rn:R03361) (1 rn:R03427) (1 rn:R03430) (1 rn:R03433) (1 rn:R03435) (1 rn:R03469) (1 rn:R04372) (1 rn:R07377)	3 CTP + L-Ser = CDP-EtOHamine + 2 CMP
33	13	false	13	(1 rn:R01021) (1 rn:R01321) (1 rn:R01890) (1 rn:R02053) (1 rn:R02055) (1 rn:R03361) (1 rn:R03416) (1 rn:R03435) (1 rn:R03469) (-1 rn:R05202) (1 rn:R05800) (1 rn:R05801) (1 rn:R07377)	CTP + L-Ser + PI = CMP + 2 Fattyacid + inositolHP + sn-glycero-3-PEtOHamine
34	16	false	16	(1 rn:R01021) (1 rn:R01184) (1 rn:R01187) (1 rn:R01321) (1 rn:R01890) (1 rn:R02053) (1 rn:R02055) (1 rn:R03361) (1 rn:R03416) (1 rn:R03427) (1 rn:R03430) (1 rn:R03433) (1 rn:R03435) (1 rn:R03469) (1 rn:R04372) (1 rn:R07377)	CTP + L-Ser + Oxygen + PI = CMP + D-Glucuronate + 2 Fattyacid + sn-glycero-3-PEtOHamine
35	9	false	9	(1 rn:R01315) (1 rn:R01321) (1 rn:R02746) (1 rn:R03361) (1 rn:R03435) (1 rn:R03469) (-1 rn:R05202) (1 rn:R05800) (1 rn:R05801)	CDP-choline + PI = CMP + 2 Fattyacid + inositolHP + sn-glycero-3-Pcholine
36	12	false	12	(1 rn:R01184) (1 rn:R01187) (1 rn:R01315) (1 rn:R01321) (1 rn:R02746) (1 rn:R03361) (1 rn:R03427) (1 rn:R03430) (1 rn:R03433) (1 rn:R03435) (1 rn:R03469) (1 rn:R04372)	CDP-choline + Oxygen + PI = CMP + D-Glucuronate + 2 Fattyacid + sn-glycero-3-Pcholine
37	12	false	12	(1 rn:R01021) (1 rn:R01184) (1 rn:R01186) (1 rn:R01310) (1 rn:R01321) (1 rn:R01890) (1 rn:R02241) (1 rn:R03361) (1 rn:R03393) (1 rn:R03394) (1 rn:R03435) (1 rn:R03469)	CTP + CoA + Oxygen + PI = 1-Acyl-sn-glycerol3-P + Acyl-CoA + CMP + D-Glucuronate
38	13	false	13	(1 rn:R01021) (1 rn:R01184) (1 rn:R01186) (1 rn:R01310) (1 rn:R01321) (1 rn:R01799) (1 rn:R01890) (1 rn:R02030) (1 rn:R03361) (1 rn:R03393) (1 rn:R03394) (1 rn:R03435) (1 rn:R03469)	2 CTP + Oxygen + PI + Phosphatidylglycerol = 2 CMP + Cardiolipin + D-Glucuronate
39	12	false	12	(1 rn:R01021) (1 rn:R01186) (1 rn:R01310) (1 rn:R01321) (1 rn:R01799) (1 rn:R01802) (1 rn:R01890) (1 rn:R03361) (1 rn:R03393) (1 rn:R03394) (1 rn:R03435) (1 rn:R03469)	2 CTP = 2 CMP
40	13	false	13	(1 rn:R01021) (1 rn:R01184) (1 rn:R01186) (1 rn:R01321) (1 rn:R01890) (1 rn:R02055) (1 rn:R02056) (1 rn:R03361) (1 rn:R03393) (1 rn:R03394) (1 rn:R03435) (1 rn:R03469) (1 rn:R07377)	Adenosyl-L-Met + CTP + L-Ser + Oxygen + PI = Adenosyl-L-homoCys + CMP + D-Glucuronate + Phosphatidyl-N-methylEtOHamine
41	16	false	16	(1 rn:R01021) (1 rn:R01184) (1 rn:R01186) (1 rn:R01321) (1 rn:R01468) (1 rn:R01890) (1 rn:R02038) (1 rn:R02051) (1 rn:R02055) (1 rn:R02241) (1 rn:R03361) (1 rn:R03393) (1 rn:R03394) (1 rn:R03435) (1 rn:R03469) (1 rn:R07377)	2 CTP + CoA + L-Ser + Oxygen + PI = 1-Acyl-sn-glycerol3-P + Acyl-CoA + CDP-EtOHamine + CMP + D-Glucuronate
42	17	false	17	(1 rn:R01021) (1 rn:R01184) (1 rn:R01186) (1 rn:R01321) (1 rn:R01468) (1 rn:R01799) (1 rn:R01890) (1 rn:R02030) (1 rn:R02038) (1 rn:R02051) (1 rn:R02055) (1 rn:R03361) (1 rn:R03393) (1 rn:R03394) (1 rn:R03435) (1 rn:R03469) (1 rn:R07377)	3 CTP + L-Ser + Oxygen + PI + Phosphatidylglycerol = CDP-EtOHamine + 2 CMP + Cardiolipin + D-Glucuronate

43	16	false	16	(1 rn:R01021) (1 rn:R01186) (1 rn:R01321) (1 rn:R01468) (1 rn:R01799) (1 rn:R01802) (1 rn:R01890) (1 rn:R02038) (1 rn:R02051) (1 rn:R02055) (1 rn:R03361) (1 rn:R03393) (1 rn:R03394) (1 rn:R03435) (1 rn:R03469) (1 rn:R07377)	3 CTP + L-Ser = CDP-EtOHamine + 2 CMP
44	14	false	14	(1 rn:R01021) (1 rn:R01184) (1 rn:R01186) (1 rn:R01321) (1 rn:R01890) (1 rn:R02053) (1 rn:R02055) (1 rn:R03361) (1 rn:R03393) (1 rn:R03394) (1 rn:R03416) (1 rn:R03435) (1 rn:R03469) (1 rn:R07377)	CTP + L-Ser + Oxygen + PI = CMP + D-Glucuronate + 2 Fattyacid + sn-glycero-3-PEtOHamine
45	10	false	10	(1 rn:R01184) (1 rn:R01186) (1 rn:R01315) (1 rn:R01321) (1 rn:R02746) (1 rn:R03361) (1 rn:R03393) (1 rn:R03394) (1 rn:R03435) (1 rn:R03469)	CDP-choline + Oxygen + PI = CMP + D-Glucuronate + 2 Fattyacid + sn-glycero-3-Pcholine
46	8	false	8	(1 rn:R02240) (1 rn:R02241) (1 rn:R03361) (1 rn:R03435) (1 rn:R03469) (-1 rn:R05202) (1 rn:R05800) (1 rn:R05801)	CoA + PI = 1-Acyl-sn-glycerol3-P + Acyl-CoA + inositolHP
47	9	false	9	(1 rn:R01799) (1 rn:R02030) (1 rn:R02240) (1 rn:R03361) (1 rn:R03435) (1 rn:R03469) (-1 rn:R05202) (1 rn:R05800) (1 rn:R05801)	CTP + PI + Phosphatidylglycerol = CMP + Cardiolipin + inositolHP
48	11	false	11	(-1 rn:R00840) (1 rn:R01185) (1 rn:R01799) (1 rn:R01802) (1 rn:R02240) (1 rn:R03361) (1 rn:R03435) (1 rn:R03469) (-1 rn:R05202) (1 rn:R05800) (1 rn:R05801)	CTP + G6P = CMP + inositolHP
49	11	false	11	(1 rn:R01184) (1 rn:R01187) (1 rn:R02240) (1 rn:R02241) (1 rn:R03361) (1 rn:R03427) (1 rn:R03430) (1 rn:R03433) (1 rn:R03435) (1 rn:R03469) (1 rn:R04372)	CoA + Oxygen + PI = 1-Acyl-sn-glycerol3-P + Acyl-CoA + D-Glucuronate
50	12	false	12	(1 rn:R01184) (1 rn:R01187) (1 rn:R01799) (1 rn:R02030) (1 rn:R02240) (1 rn:R03361) (1 rn:R03427) (1 rn:R03430) (1 rn:R03433) (1 rn:R03435) (1 rn:R03469) (1 rn:R04372)	CTP + Oxygen + PI + Phosphatidylglycerol = CMP + Cardiolipin + D-Glucuronate
51	11	false	11	(1 rn:R01187) (1 rn:R01799) (1 rn:R01802) (1 rn:R02240) (1 rn:R03361) (1 rn:R03427) (1 rn:R03430) (1 rn:R03433) (1 rn:R03435) (1 rn:R03469) (1 rn:R04372)	CTP = CMP
52	9	false	9	(1 rn:R01184) (1 rn:R01186) (1 rn:R02240) (1 rn:R02241) (1 rn:R03361) (1 rn:R03393) (1 rn:R03394) (1 rn:R03435) (1 rn:R03469)	CoA + Oxygen + PI = 1-Acyl-sn-glycerol3-P + Acyl-CoA + D-Glucuronate
53	10	false	10	(1 rn:R01184) (1 rn:R01186) (1 rn:R01799) (1 rn:R02030) (1 rn:R02240) (1 rn:R03361) (1 rn:R03393) (1 rn:R03394) (1 rn:R03435) (1 rn:R03469)	CTP + Oxygen + PI + Phosphatidylglycerol = CMP + Cardiolipin + D-Glucuronate
54	9	false	9	(1 rn:R01186) (1 rn:R01799) (1 rn:R01802) (1 rn:R02240) (1 rn:R03361) (1 rn:R03393) (1 rn:R03394) (1 rn:R03435) (1 rn:R03469)	CTP = CMP
55	9	false	9	(1 rn:R01321) (1 rn:R02114) (1 rn:R02746) (1 rn:R03361) (1 rn:R03435) (1 rn:R03469) (-1 rn:R05202) (1 rn:R05800) (1 rn:R05801)	CDP-choline + PI + Sterol = CMP + Fattyacid + Sterylester + inositolHP + sn-glycero-3-Pcholine

56	12	false	12	(1 rn:R01184) (1 rn:R01187) (1 rn:R01321) (1 rn:R02114) (1 rn:R02746) (1 rn:R03361) (1 rn:R03427) (1 rn:R03430) (1 rn:R03433) (1 rn:R03435) (1 rn:R03469) (1 rn:R04372)	CDP-choline + Oxygen + PI + Sterol = CMP + D-Glucuronate + Fattyacid + Sterylester + sn-glycero-3-Pcholine
57	10	false	10	(1 rn:R01184) (1 rn:R01186) (1 rn:R01321) (1 rn:R02114) (1 rn:R02746) (1 rn:R03361) (1 rn:R03393) (1 rn:R03394) (1 rn:R03435) (1 rn:R03469)	CDP-choline + Oxygen + PI + Sterol = CMP + D-Glucuronate + Fattyacid + Sterylester + sn-glycero-3-Pcholine

Table 7: *S. saprophyticus* network - metabolites

Name	internal?	Connectivity	Description
(5-P-D-R)anthranilate	true	2	N-(5-Phospho-D-ribosyl)anthranilate
1-(2-Carboxyphenylamino)-1-deoxy-D-Ru_5-P	true	2	1-(2-Carboxyphenylamino)-1-deoxy-D-ribulose_5-phosphate
2-(A-Hydroxyethyl)thiamine_diP	true	3	2-(alpha-Hydroxyethyl)thiamine_diphosphate
2-Oxobutanoate	true	2	2-Oxobutanoate
2-P-D-GA	true	2	2-Phospho-D-glycerate
23-Dihydrodipicolinate	true	2	2,3-Dihydrodipicolinate
2345-Tetrahydrodipicolinate	true	2	2,3,4,5-Tetrahydrodipicolinate
3-(4-Hydroxyphenyl)PYR	true	2	3-(4-Hydroxyphenyl)pyruvate
3-(Imidazol-4-yl)-2-oxopropyl_P	true	2	3-(Imidazol-4-yl)-2-oxopropyl_phosphate
3-DehydroShi	true	2	3-Dehydroshikimate
3-Methyl-2-oxobutanoate	true	3	3-Methyl-2-oxobutanoate
3-P-D-GA	true	2	3-Phospho-D-glycerate
3-P-D-glyceroyl_P	true	2	3-Phospho-D-glyceroyl_phosphate
4-Imidazolone-5-propanoate	true	3	4-Imidazolone-5-propanoate
4-P-L-Asp	true	2	4-Phospho-L-aspartate
5-O-(1-Carboxyvinyl)-3-PShi	true	2	5-O-(1-Carboxyvinyl)-3-phosphoshikimate
510-MethyleneTHF	true	2	5,10-Methylenetetrahydrofolate
6PGlucon	true	2	6-Phospho-D-gluconate
Ac	true	4	Acetate
Ac-CoA	true	6	Acetyl-CoA
Acetaldehyde	true	1	Acetaldehyde
AICAR	true	2	1-(5-Phosphoribosyl)-5-amino-4-imidazolecarboxamide
AKG	true	14	2-Oxoglutarate
Ala-tRNA(ex)	false	1	Ala-tRNA(ex)
Anthranilate	true	2	Anthranilate
Arg-tRNA(ex)	false	1	Arg-tRNA(ex)
Asn-tRNA(ex)	false	1	Asn-tRNA(ex)
Asp-tRNA(ex)	false	1	Asp-tRNA(ex)
Carbamoyl_P	true	2	Carbamoyl_phosphate
Chor	true	3	Chorismate
cis-Acon	true	2	cis-Aconitate
Cit	true	2	Citrate
CoA	true	9	CoA
Cys-tRNA(ex)	false	1	Cys-tRNA(ex)
D-Ala	true	2	D-Alanine
D-E4P	true	3	D-Erythrose_4-phosphate
D-erythro-1-(Imidazol-4-yl)glycerol_3-P	true	2	D-erythro-1-(Imidazol-4-yl)glycerol_3-phosphate

D-F	true	2	D-Fructose
D-F_16-BP	true	3	D-Fructose_1,6-bisphosphate
D-F_6-P	true	6	D-Fructose_6-phosphate
D-G(ex)	false	1	D-Glucose(ex)
D-G_6-P	true	4	D-Glucose_6-phosphate
D-Glu	true	2	D-Glutamate
D-Glucono-15-lactone_6-P	true	2	D-Glucono-1,5-lactone_6-phosphate
D-Ri_5-P	true	3	D-Ribose_5-phosphate
D-Ru_5-P	true	3	D-Ribulose_5-phosphate
D-S7P	true	2	D-Sedoheptulose_7-phosphate
D-Xu_5-P	true	3	D-Xylulose_5-phosphate
DAHP	true	2	2-Dehydro-3-deoxy-D-arabino-heptonate_7-phosphate
DHQ	true	2	3-Dehydroquinate
FAD	false	1	FAD
FADH2	false	1	FADH2
Formamide	true	1	Formamide
Formimino-L-Glu	true	2	N-Formimino-L-glutamate
Fum	true	3	Fumarate
GA_P	true	7	(2R)-2-Hydroxy-3-(phosphonooxy)-propanal
Gln-tRNA(ex)	false	1	Gln-tRNA(ex)
Glu-tRNA(ex)	false	1	Glu-tRNA(ex)
Gly	true	3	Glycine
Gly-tRNA(ex)	false	1	Gly-tRNA(ex)
Glycerone_P	true	2	Glycerone_phosphate
His-tRNA(ex)	false	1	His-tRNA(ex)
Ile-tRNA(ex)	false	1	Ile-tRNA(ex)
Indoleglycerol_P	true	2	Indoleglycerol_phosphate
IsoCit	true	2	Isocitrate
L-Ala	true	2	L-Alanine
L-Arg	true	4	L-Arginine
L-Asn	true	2	L-Asparagine
L-Asp	true	5	L-Aspartate
L-Asp_4-semialdehyde	true	3	L-Aspartate_4-semialdehyde
L-Citrulline	true	3	L-Citrulline
L-Cys	true	3	L-Cysteine
L-Gln	true	5	L-Glutamine
L-Glu	true	17	L-Glutamate
L-His	true	3	L-Histidine
L-Histidinol	true	2	L-Histidinol
L-Histidinol_P	true	2	L-Histidinol_phosphate

L-HomoCys	true	2	L-Homocysteine
L-HomoSer	true	3	L-Homoserine
L-Ile	true	2	L-Isoleucine
L-Leu	true	2	L-Leucine
L-Lys	true	2	L-Lysine
L-Met	true	2	L-Methionine
L-Orn	true	5	L-Ornithine
L-PhenylAla	true	2	L-Phenylalanine
L-Pro	true	3	L-Proline
L-Ser	true	5	L-Serine
L-Thr	true	3	L-Threonine
L-Try	true	2	L-Tryptophane
L-Tyr	true	2	L-Tyrosine
L-Val	true	2	L-Valine
Leu-tRNA(ex)	false	1	Leu-tRNA(ex)
Lys-tRNA(ex)	false	1	Lys-tRNA(ex)
Mal	true	3	(S)-Malate
Met-tRNA(ex)	false	1	Met-tRNA(ex)
NAD+	false	12	NAD+
NADH	false	12	NADH
NADP+	true	11	NADP+
NADPH	true	11	NADPH
NH3	true	11	NH3
NH3(ex)	false	1	NH3(ex)
O-Ac-L-Ser	true	2	O-Acetyl-L-serine
O-P-L-homoSer	true	2	O-Phospho-L-homoserine
OAA	true	5	Oxaloacetate
OASucc	true	2	Oxalosuccinate
PEP	true	7	Phosphoenolpyruvate
Phe-tRNA(ex)	false	1	Phe-tRNA(ex)
PhenylPYR	true	2	Phenylpyruvate
PR-AMP	true	2	Phosphoribosyl-AMP
PR-ATP	true	2	Phosphoribosyl-ATP
PR-formimino-AICAR-P	true	2	5-(5-Phospho-D-ribosylaminoformimino)-1-(5-phosphoribosyl)-imidazole-4-carboxamide
Prephenate	true	3	Prephenate
Pribulosyl-formimino-AICAR-P	true	2	N-(5-Phospho-D-1-ribulosylformimino)-5-amino-1-(5-phospho-D-ribosyl)-4-imidazolecarboxamide
Pro-tRNA(ex)	false	1	Pro-tRNA(ex)
PRPP	true	4	5-Phospho-D-ribose_1-diphosphate
Purine(ex)	false	1	Purine(ex)

PYR	true	13	Pyruvate
Pyrimidine(ex)	false	1	Pyrimidine(ex)
Ser-tRNA(ex)	false	1	Ser-tRNA(ex)
Shi	true	2	Shikimate
Shi_3-P	true	2	Shikimate_3-phosphate
Succ	true	3	Succinate
SuccCoA	true	3	Succinyl-CoA
Sucrose_6-P	true	1	Sucrose_6-phosphate
THF	true	2	Tetrahydrofolate
Thiamin_diP	true	3	Thiamin_diphosphate
Thr-tRNA(ex)	false	1	Thr-tRNA(ex)
Try-tRNA(ex)	false	1	Try-tRNA(ex)
Tyr-tRNA(ex)	false	1	Tyr-tRNA(ex)
Urea	true	2	Urea
Urocanate	true	2	Urocanate
Val-tRNA(ex)	false	1	Val-tRNA(ex)

Table 8: *S. saprophyticus* network - reactions

Enzyme name	rev.?	Reaction equation	Annotation
rn:E0001	false	L-PhenylAla = Phe-tRNA(ex)	PheX [6.1.1.20]
rn:E0002	false	L-Tyr = Tyr-tRNA(ex)	TyrX [6.1.1.1]
rn:E0003	false	L-Try = Try-tRNA(ex)	TryX [6.1.1.-]
rn:E0004	false	L-His = His-tRNA(ex)	HisX [6.1.1.21]
rn:E0005	false	L-Met = Met-tRNA(ex)	MetX [6.1.1.10]
rn:E0006	false	L-Thr = Thr-tRNA(ex)	ThrX [6.1.1.3]
rn:E0007	false	Gly = Gly-tRNA(ex)	GlyX [6.1.1.14]
rn:E0008	false	L-Ser = Ser-tRNA(ex)	SerX [6.1.1.11]
rn:E0009	false	L-Cys = Cys-tRNA(ex)	CysX [6.1.1.16]
rn:E0010	false	L-Lys = Lys-tRNA(ex)	LysX [6.1.1.6]
rn:E0011	false	L-Asn = Asn-tRNA(ex)	AsnX [6.1.1.22]
rn:E0012	false	L-Asp = Asp-tRNA(ex)	AspX [6.1.1.12]
rn:E0013	false	L-Ala = Ala-tRNA(ex)	AlaX [6.1.1.7]
rn:E0014	false	L-Pro = Pro-tRNA(ex)	ProX [6.1.1.15]
rn:E0015	false	L-Arg = Arg-tRNA(ex)	ArgX [6.1.1.19]
rn:E0016	false	L-Glu = Glu-tRNA(ex)	GluX [6.1.1.17]
rn:E0017	false	L-Gln = Gln-tRNA(ex)	GlnX [6.1.1.17]
rn:E0018	false	L-Ile = Ile-tRNA(ex)	IleX [6.1.1.5]
rn:E0019	false	L-Val = Val-tRNA(ex)	ValX [6.1.1.9]
rn:E0020	false	L-Leu = Leu-tRNA(ex)	LeuX [6.1.1.4]
rn:N001	false	AICAR = Purine(ex)	PurineMeta []
rn:N002	false	PRPP = Pyrimidine(ex)	PyrimidineMeta []
rn:R001	false	PYR = PEP	PPDK [2.7.9.1]
rn:R002	false	4-Imidazolone-5-propanoate = 2 L-Glu	HisMetaEnzOxReDase [1.14.13.-]
rn:U0015	false	1-(2-Carboxyphenylamino)-1-deoxy-D-Ru_5-P = Indoleglycerol_P	indole-3-glycerolphosphatesynthase [4.1.1.48]
rn:U0018	false	L-Gln = Carbamoyl_P + L-Glu	carbamoyl-phosphatesynthase [6.3.5.5]
rn:U0020	false	23-Dihydrodipicolinate + NADPH = 2345-Tetrahydrodipicolinate + NADP+	dihydrodipicolinate reductase [1.3.1.26]
rn:U0021	false	DAHP = DHQ	3-dehydroquinatesynthase [4.2.3.4]
rn:U0038	false	3-Methyl-2-oxobutanoate + L-Glu = AKG + L-Val	branched-chainaminoacidaminotransferase [2.6.1.42]
rn:U0045	false	4-Imidazolone-5-propanoate = Formimino-L-Glu	imidazolonepropionase [3.5.2.7]
rn:U0047	false	4-P-L-Asp + NADPH = L-Asp_4-semialdehyde + NADP+	aspartate semialdehyde dehydrogenase [1.2.1.11]
rn:U0048	false	PR-formimino-AICAR-P = Pribulosyl-formimino-AICAR-P	putative phosphoribosylformimino-5-aminoimidazolecarboxamideri-botideisomerase [5.3.1.16]
rn:U0049	false	510-MethyleneTHF + Gly = L-Ser + THF	serinehydroxymethyltransferase [2.1.2.1]

rn:U0050	false	L-HomoCys = L-Met	5-methyltetrahydropteroyltriglutamate-homocysteinemethyltrans-ferase [2.1.1.14]
rn:U0052	false	5-O-(1-Carboxyvinyl)-3-PShi = Chor	chorismatesynthase [4.2.3.5]
rn:U0057	false	Anthranilate + PRPP = (5-P-D-R)anthranilate	anthranilatephosphoribosyltransferase [2.4.2.18]
rn:U0058	false	L-Asp + L-Gln = L-Asn + L-Glu	asparagine synthase [6.3.5.4]
rn:U0059	false	L-Asp = 4-P-L-Asp	asparto kinase II [2.7.2.4]
rn:U0061	false	L-Glu + NH3 = L-Gln	glutamine-ammonialigase [6.3.1.2]
rn:U0062	false	L-HomoSer = O-P-L-homoSer	homoserinekinase [2.7.1.39]
rn:U0065	false	Shi = Shi_3-P	shikimatekinase(SK) [2.7.1.71]
rn:U0068	false	Carbamoyl_P + L-Orn = L-Citrulline	ornithinecarbamoyltransferase [2.1.3.3]
rn:U0072	false	Chor + NH3 = Anthranilate + PYR	anthranilatesynthase [4.1.3.27]
rn:U0073	false	Chor = Prephenate	chorismatemutase [5.4.99.5]
rn:U0075	false	D-erythro-1-(Imidazol-4-yl)glycerol_3-P = 3-(Imidazol-4-yl)-2-oxopropyl_P	imidazoleglycerol-phosphatedehydratase [4.2.1.19]
rn:U0089	false	L-Arg = L-Citrulline + NH3	argininedeiminase [3.5.3.6]
rn:U0090	false	L-Arg = L-Orn + Urea	arginase [3.5.3.1]
rn:U0093	false	L-Glu + OAA = AKG + L-Asp	aspartate transaminase [2.6.1.1]
rn:U0095	false	L-Asp_4-semialdehyde + PYR = 23-Dihydrodipicolinate	dihydrodipicolinate synthase [4.2.1.52]
rn:U0100	true	L-Glu = D-Glu	glutamateracemase [5.1.1.3]
rn:U0104	false	L-Histidinol + 2 NAD+ = L-His + 2 NADH	putative histidinoldehydrogenase [1.1.1.23]
rn:U0105	false	L-Histidinol_P = L-Histidinol	putative histidinol phosphatase [3.1.3.15]
rn:U0106	true	L-HomoSer + NADP+ = L-Asp_4-semialdehyde + NADPH	homoserine dehydrogenase [1.1.1.3]
rn:U0112	false	L-Orn = L-Pro + NH3	putative ornithinecyclodeaminase [4.3.1.12]
rn:U0114	false	Ac-CoA + L-Ser = CoA + O-Ac-L-Ser	serineO-acetyltransferase [2.3.1.30]
rn:U0116	false	Indoleglycerol_P + L-Ser = GA_P + L-Try	tryptophan synthase [4.2.1.20]
rn:U0117	false	NH3 + PYR = L-Ser	thereoninedehydratase [4.3.1.19, 4.3.1.17]
rn:U0121	false	L-Thr = 2-Oxobutanoate + NH3	thereoninedehydratase [4.3.1.19]
rn:U0125	false	Pribulosyl-formimino-AICAR-P = AICAR + D-erythro-1-(Imidazol-4-yl)glycerol_3-P	amidotransferasehisH [2.4.2.-]
rn:U0126	false	(5-P-D-R)anthranilate = 1-(2-Carboxyphenylamino)-1-deoxy-D-Ru_5-P	phosphoriborylanthranilateisomerase [5.3.1.24]
rn:U0134	false	Formimino-L-Glu = Formamide + L-Glu	hypothetical protein,similartoformiminoglutamase [3.5.3.8]
rn:U0138	false	O-P-L-homoSer = L-Thr	threoninesynthase [4.2.3.1]
rn:U0141	false	D-E4P + PEP = DAHP	phospho-2-dehydro-3-deoxyheptonate aldo [2.5.1.54]
rn:U0142	false	PEP + Shi_3-P = 5-O-(1-Carboxyvinyl)-3-PShi	3-phosphoshikimate1-carboxyvinyltransferase [2.5.1.19]
rn:U0143	false	PR-AMP = PR-formimino-AICAR-P	histidinebiosynthesisbifunctionalproteinHisIE [3.5.4.19]
rn:U0144	false	PR-ATP = PR-AMP	histidinebiosynthesisbifunctionalproteinHisIE [3.6.1.31]
rn:U0145	false	PRPP = PR-ATP	ATPphosphoribosyltransferase [2.4.2.17]
rn:U0146	false	NAD+ + Prephenate = 3-(4-Hydroxyphenyl)PYR + NADH	prephenatedehydrogenase [1.3.1.12]

rn:U0147	false	Prephenate = PhenylPYR	prephenate dehydratase [4.2.1.51]
rn:U0155	false	3-DehydroShi + NADPH = NADP+ + Shi	shikimatedehydrogenease [1.1.1.25]
rn:U0160	false	Urea = 2 NH3	urease [3.5.1.5]
rn:U0161	false	Urocanate = 4-Imidazolone-5-propanoate	urocanatehydratase [4.2.1.49]
rn:U0166	false	3-(Imidazol-4-yl)-2-oxopropyl_P + L-Glu = AKG + L-Histidinol_P	putative histidinol-phosphateaminotransferase [2.6.1.9]
rn:U0167	false	L-Glu + PhenylPYR = AKG + L-PhenylAla	putativehistidinol-phosphateaminotransferase [2.6.1.9]
rn:U0168	false	3-(4-Hydroxyphenyl)PYR + L-Glu = AKG + L-Tyr	putative histidinol-phosphatetransaminase [2.6.1.9]
rn:U0179	false	Acetaldehyde + NAD+ = Ac + NADH	aldehyde dehydrogenase [1.2.1.3]
rn:U0188	false	DHQ = 3-DehydroShi	enolase [4.2.1.11, 4.2.1.10]
rn:U0195	false	PYR + Thiamin_diP = 2-(A-Hydroxyethyl)thiamine_diP	pyruvate dehydrogenase_acetolactatesynthase [1.2.4.1, 2.2.1.6]
rn:U0198	false	O-Ac-L-Ser = Ac + L-Cys	cysteinesynthase [2.5.1.47]
rn:U0209	false	D-Ala = L-Ala	alanineracemase [5.1.1.1]
rn:U0238	false	GA_P + NAD+ = 3-P-D-glyceroyl_P + NADH	glyceraldehyde-3-phosphate dehydrogenase [1.2.1.12]
rn:U0239	false	Glycerone_P = GA_P	triosephosphate isomerase [5.3.1.1]
rn:U0244	false	Mal + NAD+ = NADH + OAA	MDH [1.1.1.37]
rn:U0245	true	Mal + NAD+ = NADH + PYR	malatedehydrogenase homolog [1.1.1.38]
rn:U0246	false	Fum = Mal	fumarate hydratase, class-II [4.2.1.2]
rn:U0255	false	3-P-D-GA = 2-P-D-GA	phosphoglycerate mutase [5.4.2.1]
rn:U0256	false	2-P-D-GA = PEP	enolase [4.2.1.11]
rn:U0260	false	6PGlucon + NADP+ = D-Ru_5-P + NADPH	phosphogluconate dehydrogenase [1.1.1.44]
rn:U0267	false	Ac-CoA + OAA = Cit + CoA	citrate synthase [2.3.3.1]
rn:U0269	false	PEP = PYR	pyruvate kinase [2.7.1.40]
rn:U0270	false	3-P-D-glyceroyl_P = 3-P-D-GA	phosphoglycerate kinase [2.7.2.3]
rn:U0271	false	Ac + CoA = Ac-CoA	acetyl-CoA synthetase AND ligase [6.2.1.1]
rn:U0274	false	D-F = D-F_6-P	fructokinase,putative [2.7.1.4]
rn:U0276	false	D-F_6-P = D-F_16-BP	6-phosphofructokinase [2.7.1.11]
rn:U0280	false	D-Ri_5-P = PRPP	ribose-phosphate pyrophosphokinase [2.7.6.1]
rn:U0284	false	OAA = PEP	phosphoenolpyruvate carboxykinase [4.1.1.49]
rn:U0287	false	PYR = OAA	pyruvate carboxylase [6.4.1.1]
rn:U0288	true	CoA + Succ = SuccCoA	succinyl-CoA synthetase [6.2.1.5]
rn:U0294	false	D-F_16-BP = D-F_6-P	fructose-bisphosphatase [3.1.3.11]
rn:U0295	true	D-F_6-P + GA_P = D-E4P + D-Xu_5-P	transketolase [2.2.1.1]
rn:U0297	false	D-Glucono-15-lactone_6-P = 6PGlucon	Spon_PGLactonohydrolase [3.1.1.31]
rn:U0301	false	D-G_6-P + NADP+ = D-Glucono-15-lactone_6-P + NADPH	glucose-6-phosphate 1-dehydrogenase [1.1.1.49]
rn:U0302	false	D-G_6-P = D-F_6-P	glucose-6-phosphate isomerase A [5.3.1.9]
rn:U0311	false	D-Ru_5-P = D-Ri_5-P	ribose 5-phosphate isomerase A [5.3.1.6]
rn:U0314	false	D-Ru_5-P = D-Xu_5-P	ribulose-phosphate 3-epimerase [5.1.3.1]
rn:U0315	true	D-S7P + GA_P = D-E4P + D-F_6-P	Transaldolase superfamily [2.2.1.2]

rn:U0316	true	D-S7P + GA_P = D-Ri_5-P + D-Xu_5-P	transketolase [2.2.1.1]
rn:U0324	false	IsoCit + NADP+ = NADPH + OASucc	isocitrate dehyrogenase [1.1.1.42]
rn:U0335	false	OASucc = AKG	isocitrate dehyrogenase [1.1.1.42]
rn:U0345	false	FAD + Succ = FADH2 + Fum	succinate dehydrogenase [1.3.99.1]
rn:U0348	false	Sucrose_6-P = D-F + D-G_6-P	sucrose-6-phosphatehydrolase [3.2.1.26]
rn:U0358	false	cis-Acon = IsoCit	aconitate hydratase [4.2.1.3]
rn:U0359	false	Cit = cis-Acon	aconitate hydratase [4.2.1.3]
rn:U0360	false	D-F_16-BP = GA_P + Glycerone_P	fructose-bisphosphate aldolase [4.1.2.13]
rn:U0372	false	AKG + L-Gln + NADPH = 2 L-Glu + NADP+	glutamatesynthase [1.4.1.13]
rn:U0384	false	L-His = NH3 + Urocanate	histidineammonia-lyase [4.3.1.3]
rn:U0522	true	NH3(ex) = NH3	probabale ammonium transporter [2.A.49]
rn:U0534	false	D-G(ex) + PEP = D-G_6-P + PYR	glucose-specific PTS [2.7.1.69, 2.7.3.9]
rn:U0673	true	AKG + D-Ala = D-Glu + PYR	D-alanineaminotransferase [2.6.1.21]
rn:U0986	false	2 L-Glu + NADH = AKG + L-Orn + NAD+	EZComp_UreaOrnSyn [1.5.1.12, 2.6.1.13]
rn:U0987	false	2345-Tetrahydrodipicolinate + L-Glu + SuccCoA = AKG + CoA + L-Lys + Succ	EZComp_LysSyn [2.3.1.117, 2.6.1.17, 3.5.1.18, 5.1.1.7, 4.1.1.20]
rn:U0988	false	AKG + CoA + NAD+ = NADH + SuccCoA	EZComp_TCASuccinyl [1.2.4.2,2.3.1.61, 1.8.1.4]
rn:U0989	false	2-(A-Hydroxyethyl)thiamine_diP + 2-Oxobutanoate + L-Glu + NADPH = AKG + L-Ile + NADP+ + Thiamin_diP	EZComp_Ile_Meta [2.2.1.6, 1.1.1.86, 4.2.1.9, 2.6.1.42]
rn:U0990	false	2-(A-Hydroxyethyl)thiamine_diP + NADPH + PYR = 3-Methyl-2-ox-obutanoate + NADP+ + Thiamin_diP	EZComp_Val_Meta [2.2.1.6, 1.1.1.86, 4.2.1.9]
rn:U0991	false	3-Methyl-2-oxobutanoate + Ac-CoA + L-Glu + NAD+ = AKG + CoA + L-Leu + NADH	EZComp_Leu_Meta [2.3.3.13, 4.2.1.33, 1.1.1.85, 2.6.1.42]
rn:U0993	false	L-Asp + L-Citrulline = Fum + L-Arg	EZComp_Fum_Asp [4.3.2.1, 6.3.4.5]
rn:U0994	false	CoA + NAD+ + PYR = Ac-CoA + NADH	EZComp_PyrAcetylCoA [1.2.4.1, 2.3.1.12, 1.8.1.4]
rn:U0995	false	Gly + NAD+ + THF = 510-MethyleneTHF + NADH + NH3	EZComp_GlySerThrMeta [2.1.2.10, 1.8.1.4, 1.4.4.2]
rn:U0996	false	Ac-CoA + L-Cys + L-HomoSer = Ac + CoA + L-HomoCys + NH3 + PYR	EZComp_MetMeta [2.3.1.31, 2.5.1.48, 4.4.1.8]
rn:U0997	false	AKG + L-Orn + NADH + 2 NADPH = 2 L-Pro + NAD+ + 2 NADP+	EZComp_UreaMeta [1.5.1.2, 1.5.1.12, 2.6.1.13]

Table 9: *S. saprophyticus* network - Elementary Modes discussed in the results section[1]

#	Flux sum	Rev.?[2]	Length	Reactions[3]	Net reaction
4	190	false	25	(12 rn:E0012) (12 rn:U0061) (12 rn:U0093) (12 rn:U0238) (5 rn:U0239) (12 rn:U0244) (-12 rn:U0245) (12 rn:U0255) (12 rn:U0256) (6 rn:U0260) (5 rn:U0269) (12 rn:U0270) (5 rn:U0276) (-2 rn:U0295) (6 rn:U0297) (6 rn:U0301) (1 rn:U0302) (2 rn:U0311) (4 rn:U0314) (2 rn:U0315) (-2 rn:U0316) (5 rn:U0360) (12 rn:U0372) (12 rn:U0522) (7 rn:U0534)	7 D-G(ex) + 12 NAD+ + 12 NH3(ex) = 12 Asp-tRNA(ex) + 12 NADH
5	226	false	26	(12 rn:E0011) (12 rn:U0058) (24 rn:U0061) (12 rn:U0093) (12 rn:U0238) (5 rn:U0239) (12 rn:U0244) (-12 rn:U0245) (12 rn:U0255) (12 rn:U0256) (6 rn:U0260) (5 rn:U0269) (12 rn:U0270) (5 rn:U0276) (-2 rn:U0295) (6 rn:U0297) (6 rn:U0301) (1 rn:U0302) (2 rn:U0311) (4 rn:U0314) (2 rn:U0315) (-2 rn:U0316) (5 rn:U0360) (12 rn:U0372) (24 rn:U0522) (7 rn:U0534)	7 D-G(ex) + 12 NAD+ + 24 NH3(ex) = 12 Asn-tRNA(ex) + 12 NADH
6	103	false	27	(5 rn:E0008) (6 rn:U0061) (6 rn:U0089) (6 rn:U0093) (5 rn:U0117) (5 rn:U0238) (2 rn:U0239) (6 rn:U0244) (6 rn:U0246) (5 rn:U0255) (5 rn:U0256) (3 rn:U0260) (2 rn:U0269) (5 rn:U0270) (2 rn:U0276) (-1 rn:U0295) (3 rn:U0297) (3 rn:U0301) (1 rn:U0311) (2 rn:U0314) (1 rn:U0315) (-1 rn:U0316) (2 rn:U0360) (6 rn:U0372) (5 rn:U0522) (3 rn:U0534) (6 rn:U0993)	3 D-G(ex) + 11 NAD+ + 5 NH3(ex) = 11 NADH + 5 Ser-tRNA(ex)
7	88	false	27	(5 rn:E0012) (6 rn:U0061) (1 rn:U0089) (6 rn:U0093) (5 rn:U0238) (2 rn:U0239) (6 rn:U0244) (-5 rn:U0245) (1 rn:U0246) (5 rn:U0255) (5 rn:U0256) (3 rn:U0260) (2 rn:U0269) (5 rn:U0270) (2 rn:U0276) (-1 rn:U0295) (3 rn:U0297) (3 rn:U0301) (1 rn:U0311) (2 rn:U0314) (1 rn:U0315) (-1 rn:U0316) (2 rn:U0360) (6 rn:U0372) (5 rn:U0522) (3 rn:U0534) (1 rn:U0993)	3 D-G(ex) + 6 NAD+ + 5 NH3(ex) = 5 Asp-tRNA(ex) + 6 NADH
8	103	false	28	(5 rn:E0011) (5 rn:U0058) (11 rn:U0061) (1 rn:U0089) (6 rn:U0093) (5 rn:U0238) (2 rn:U0239) (6 rn:U0244) (-5 rn:U0245) (1 rn:U0246) (5 rn:U0255) (5 rn:U0256) (3 rn:U0260) (2 rn:U0269) (5 rn:U0270) (2 rn:U0276) (-1 rn:U0295) (3 rn:U0297) (3 rn:U0301) (1 rn:U0311) (2 rn:U0314) (1 rn:U0315) (-1 rn:U0316) (2 rn:U0360) (6 rn:U0372) (10 rn:U0522) (3 rn:U0534) (1 rn:U0993)	3 D-G(ex) + 6 NAD+ + 10 NH3(ex) = 5 Asn-tRNA(ex) + 6 NADH

21	188	false	32	(1 rn:E0010) (8 rn:E0011) (1 rn:U0020) (1 rn:U0047) (8 rn:U0058) (1 rn:U0059) (18 rn:U0061) (9 rn:U0093) (1 rn:U0095) (10 rn:U0238) (4 rn:U0239) (9 rn:U0244) (-9 rn:U0245) (10 rn:U0255) (10 rn:U0256) (6 rn:U0260) (4 rn:U0269) (10 rn:U0270) (4 rn:U0276) (1 rn:U0288) (-2 rn:U0295) (6 rn:U0297) (6 rn:U0301) (2 rn:U0311) (4 rn:U0314) (2 rn:U0315) (-2 rn:U0316) (4 rn:U0360) (10 rn:U0372) (18 rn:U0522) (6 rn:U0534) (1 rn:U0987)	6 D-G(ex) + 10 NAD+ + 18 NH3(ex) = 8 Asn-tRNA(ex) + Lys-tRNA(ex) + 10 NADH
22	136	false	34	(1 rn:E0010) (4 rn:E0019) (1 rn:U0020) (4 rn:U0038) (1 rn:U0047) (1 rn:U0059) (6 rn:U0061) (1 rn:U0093) (1 rn:U0095) (4 rn:U0195) (10 rn:U0238) (4 rn:U0239) (1 rn:U0244) (-1 rn:U0245) (10 rn:U0255) (10 rn:U0256) (6 rn:U0260) (4 rn:U0269) (10 rn:U0270) (4 rn:U0276) (1 rn:U0288) (-2 rn:U0295) (6 rn:U0297) (6 rn:U0301) (2 rn:U0311) (4 rn:U0314) (2 rn:U0315) (-2 rn:U0316) (4 rn:U0360) (6 rn:U0372) (6 rn:U0522) (6 rn:U0534) (1 rn:U0987) (4 rn:U0990)	6 D-G(ex) + 10 NAD+ + 6 NH3(ex) = Lys-tRNA(ex) + 10 NADH + 4 Val-tRNA(ex)
24	88	false	29	(5 rn:E0013) (6 rn:U0061) (1 rn:U0089) (1 rn:U0093) (5 rn:U0100) (5 rn:U0209) (5 rn:U0238) (2 rn:U0239) (1 rn:U0244) (1 rn:U0246) (5 rn:U0255) (5 rn:U0256) (3 rn:U0260) (2 rn:U0269) (5 rn:U0270) (2 rn:U0276) (-1 rn:U0295) (3 rn:U0297) (3 rn:U0301) (1 rn:U0311) (2 rn:U0314) (1 rn:U0315) (-1 rn:U0316) (2 rn:U0360) (6 rn:U0372) (5 rn:U0522) (3 rn:U0534) (-5 rn:U0673) (1 rn:U0993)	3 D-G(ex) + 6 NAD+ + 5 NH3(ex) = 5 Ala-tRNA(ex) + 6 NADH

[1] *complete list availabe from the authors on request*

[2] *reversible*

[3] *reaction identifiers are taken from KEGG, for annotations see the reaction table*

Table 10: *S. aureus* network - metabolites

Name	internal?	Connectivity	Description
(5-P-D-R)anthranilate	true	2	N-(5-Phospho-D-ribosyl)anthranilate
1-(2-Carboxyphenylamino)-1-deoxy-D-Ru_5-P	true	2	1-(2-Carboxyphenylamino)-1-deoxy-D-ribulose_5-phosphate
2-(A-Hydroxyethyl)thiamine_diP	true	3	2-(alpha-Hydroxyethyl)thiamine_diphosphate
2-Oxobutanoate	true	2	2-Oxobutanoate
2-P-D-GA	true	2	2-Phospho-D-glycerate
23-Dihydrodipicolinate	true	2	2,3-Dihydrodipicolinate
2345-Tetrahydrodipicolinate	true	2	2,3,4,5-Tetrahydrodipicolinate
3-(4-Hydroxyphenyl)PYR	true	2	3-(4-Hydroxyphenyl)pyruvate
3-(Imidazol-4-yl)-2-oxopropyl_P	true	2	3-(Imidazol-4-yl)-2-oxopropyl_phosphate
3-DehydroShi	true	2	3-Dehydroshikimate
3-Methyl-2-oxobutanoate	true	3	3-Methyl-2-oxobutanoate
3-P-D-GA	true	2	3-Phospho-D-glycerate
3-P-D-glyceroyl_P	true	2	3-Phospho-D-glyceroyl_phosphate
4-Imidazolone-5-propanoate	true	2	4-Imidazolone-5-propanoate
4-P-L-Asp	true	2	4-Phospho-L-aspartate
5-O-(1-Carboxyvinyl)-3-PShi	true	2	5-O-(1-Carboxyvinyl)-3-phosphoshikimate
510-MethyleneTHF	true	2	5,10-Methylenetetrahydrofolate
6PGlucon	true	2	6-Phospho-D-gluconate
Ac	true	4	Acetate
Ac-CoA	true	6	Acetyl-CoA
Acetaldehyde	true	2	Acetaldehyde
AICAR	true	2	1-(5-Phosphoribosyl)-5-amino-4-imidazolecarboxamide
AKG	true	14	2-Oxoglutarate
Ala-tRNA(ex)	false	1	Ala-tRNA(ex)
Anthranilate	true	2	Anthranilate
Arg-tRNA(ex)	false	1	Arg-tRNA(ex)
Asn-tRNA(ex)	false	1	Asn-tRNA(ex)
Asp-tRNA(ex)	false	1	Asp-tRNA(ex)
Carbamoyl_P	true	3	Carbamoyl_phosphate
Chor	true	3	Chorismate
cis-Acon	true	2	cis-Aconitate
Cit	true	2	Citrate
CoA	true	9	CoA
Cys-tRNA(ex)	false	1	Cys-tRNA(ex)
D-Ala	true	2	D-Alanine
D-E4P	true	3	D-Erythrose_4-phosphate
D-erythro-1-(Imidazol-4-yl)glycerol_3-P	true	2	D-erythro-1-(Imidazol-4-yl)glycerol_3-phosphate

D-F	true	2	D-Fructose
D-F_16-BP	true	3	D-Fructose_1,6-bisphosphate
D-F_6-P	true	6	D-Fructose_6-phosphate
D-G(ex)	false	1	D-Glucose(ex)
D-G_6-P	true	4	D-Glucose_6-phosphate
D-Glu	true	2	D-Glutamate
D-Glucono-15-lactone_6-P	true	2	D-Glucono-1,5-lactone_6-phosphate
D-Ri_5-P	true	3	D-Ribose_5-phosphate
D-Ru_5-P	true	3	D-Ribulose_5-phosphate
D-S7P	true	2	D-Sedoheptulose_7-phosphate
D-Xu_5-P	true	3	D-Xylulose_5-phosphate
DAHP	true	2	2-Dehydro-3-deoxy-D-arabino-heptonate_7-phosphate
DHQ	true	2	3-Dehydroquinate
FAD	false	1	FAD
FADH2	false	1	FADH2
Formamide	true	1	Formamide
Formimino-L-Glu	true	2	N-Formimino-L-glutamate
Fum	true	3	Fumarate
GA_P	true	7	(2R)-2-Hydroxy-3-(phosphonooxy)-propanal
Gln-tRNA(ex)	false	1	Gln-tRNA(ex)
Glu-tRNA(ex)	false	1	Glu-tRNA(ex)
Gly	true	4	Glycine
Gly-tRNA(ex)	false	1	Gly-tRNA(ex)
Glycerone_P	true	2	Glycerone_phosphate
His-tRNA(ex)	false	1	His-tRNA(ex)
Ile-tRNA(ex)	false	1	Ile-tRNA(ex)
Indoleglycerol_P	true	2	Indoleglycerol_phosphate
IsoCit	true	2	Isocitrate
L-Ala	true	2	L-Alanine
L-Arg	true	4	L-Arginine
L-Asn	true	2	L-Asparagine
L-Asp	true	5	L-Aspartate
L-Asp_4-semialdehyde	true	3	L-Aspartate_4-semialdehyde
L-Citrulline	true	3	L-Citrulline
L-Cys	true	3	L-Cysteine
L-Gln	true	5	L-Glutamine
L-Glu	true	16	L-Glutamate
L-His	true	3	L-Histidine
L-Histidinol	true	2	L-Histidinol
L-Histidinol_P	true	2	L-Histidinol_phosphate

L-HomoCys	true	2	L-Homocysteine
L-HomoSer	true	3	L-Homoserine
L-Ile	true	2	L-Isoleucine
L-Leu	true	2	L-Leucine
L-Lys	true	2	L-Lysine
L-Met	true	2	L-Methionine
L-Orn	true	5	L-Ornithine
L-PhenylAla	true	2	L-Phenylalanine
L-Pro	true	3	L-Proline
L-Ser	true	5	L-Serine
L-Thr	true	4	L-Threonine
L-Try	true	2	L-Tryptophane
L-Tyr	true	2	L-Tyrosine
L-Val	true	2	L-Valine
Leu-tRNA(ex)	false	1	Leu-tRNA(ex)
Lys-tRNA(ex)	false	1	Lys-tRNA(ex)
Mal	true	3	(S)-Malate
Menaquinol	true	1	Menaquinol
Menaquinone	true	1	Menaquinone
Met-tRNA(ex)	false	1	Met-tRNA(ex)
NAD+	false	11	NAD+
NADH	false	11	NADH
NADP+	true	11	NADP+
NADPH	true	11	NADPH
NH3	true	12	NH3
NH3(ex)	false	1	NH3(ex)
O-Ac-L-Ser	true	2	O-Acetyl-L-serine
O-P-L-homoSer	true	2	O-Phospho-L-homoserine
OAA	true	5	Oxaloacetate
OASucc	true	2	Oxalosuccinate
PEP	true	6	Phosphoenolpyruvate
Phe-tRNA(ex)	false	1	Phe-tRNA(ex)
PhenylPYR	true	2	Phenylpyruvate
PR-AMP	true	2	Phosphoribosyl-AMP
PR-ATP	true	2	Phosphoribosyl-ATP
PR-formimino-AICAR-P	true	2	5-(5-Phospho-D-ribosylaminoformimino)-1-(5-phosphoribosyl)-imidazole-4-carboxamide
Prephenate	true	3	Prephenate
Pribulosyl-formimino-AICAR-P	true	2	N-(5-Phospho-D-1-ribulosylformimino)-5-amino-1-(5-phospho-D-ribosyl)-4-imidazolecarboxamide
Pro-tRNA(ex)	false	1	Pro-tRNA(ex)
PRPP	true	4	5-Phospho-D-ribose_1-diphosphate

Purine(ex)	false	1	Purine(ex)
PYR	true	12	Pyruvate
Pyrimidine(ex)	false	1	Pyrimidine(ex)
Ser-tRNA(ex)	false	1	Ser-tRNA(ex)
Shi	true	2	Shikimate
Shi_3-P	true	2	Shikimate_3-phosphate
Succ	true	3	Succinate
SuccCoA	true	3	Succinyl-CoA
Sucrose_6-P	true	1	Sucrose_6-phosphate
THF	true	2	Tetrahydrofolate
Thiamin_diP	true	3	Thiamin_diphosphate
Thr-tRNA(ex)	false	1	Thr-tRNA(ex)
Try-tRNA(ex)	false	1	Try-tRNA(ex)
Tyr-tRNA(ex)	false	1	Tyr-tRNA(ex)
Urea	true	2	Urea
Urocanate	true	2	Urocanate
Val-tRNA(ex)	false	1	Val-tRNA(ex)

Table 11: *S. aureus* network - reactions

Enzyme name	rev.?	Reaction equation	Annotation
RN:E0001	false	L-PhenylAla = Phe-tRNA(ex)	PheX [6.1.1.20]
RN:E0002	false	L-Tyr = Tyr-tRNA(ex)	TyrX [6.1.1.1]
RN:E0003	false	L-Try = Try-tRNA(ex)	TryX [6.1.1.-]
RN:E0004	false	L-His = His-tRNA(ex)	HisX [6.1.1.21]
RN:E0005	false	L-Met = Met-tRNA(ex)	MetX [6.1.1.10]
RN:E0006	false	L-Thr = Thr-tRNA(ex)	ThrX [6.1.1.3]
RN:E0007	false	Gly = Gly-tRNA(ex)	GlyX [6.1.1.14]
RN:E0008	false	L-Ser = Ser-tRNA(ex)	SerX [6.1.1.11]
RN:E0009	false	L-Cys = Cys-tRNA(ex)	CysX [6.1.1.16]
RN:E0010	false	L-Lys = Lys-tRNA(ex)	LysX [6.1.1.6]
RN:E0011	false	L-Asn = Asn-tRNA(ex)	AsnX [6.1.1.22]
RN:E0012	false	L-Asp = Asp-tRNA(ex)	AspX [6.1.1.12]
RN:E0013	false	L-Ala = Ala-tRNA(ex)	AlaX [6.1.1.7]
RN:E0014	false	L-Pro = Pro-tRNA(ex)	ProX [6.1.1.15]
RN:E0015	false	L-Arg = Arg-tRNA(ex)	ArgX [6.1.1.19]
RN:E0016	false	L-Glu = Glu-tRNA(ex)	GluX [6.1.1.17]
RN:E0017	false	L-Gln = Gln-tRNA(ex)	GlnX [6.1.1.17]
RN:E0018	false	L-Ile = Ile-tRNA(ex)	IleX [6.1.1.5]
RN:E0019	false	L-Val = Val-tRNA(ex)	ValX [6.1.1.9]
RN:E0020	false	L-Leu = Leu-tRNA(ex)	LeuX [6.1.1.4]
RN:Nucl01	false	AICAR = Purine(ex)	PurineMeta []
RN:Nucl02	false	PRPP = Pyrimidine(ex)	PyrimidineMeta []
RN:R0015	false	1-(2-Carboxyphenylamino)-1-deoxy-D-Ru_5-P = Indoleglycerol_P	indole-3-glycerolphosphatesynthase [4.1.1.48]
RN:R0018	false	L-Gln = Carbamoyl_P + L-Glu	carbamoyl-phosphatesynthase [6.3.5.5]
RN:R0020	false	23-Dihydrodipicolinate + NADPH = 2345-Tetrahydrodipicolinate + NADP+	dihydrodipicolinate reductase [1.3.1.26]
RN:R0021	false	DAHP = DHQ	3-dehydroquinatesynthase [4.2.3.4]
RN:R0038	false	3-Methyl-2-oxobutanoate + L-Glu = AKG + L-Val	branched-chainaminoacidaminotransferase [2.6.1.42]
RN:R0045	false	4-Imidazolone-5-propanoate = Formimino-L-Glu	imidazolonepropionase [3.5.2.7]
RN:R0047	false	4-P-L-Asp + NADPH = L-Asp_4-semialdehyde + NADP+	aspartate semialdehyde dehydrogenase [1.2.1.11]
RN:R0048	false	PR-formimino-AICAR-P = Pribulosyl-formimino-AICAR-P	putative phosphoribosylformimino-5-aminoimidazolecarboxamideriboti-deisomerase [5.3.1.16]
RN:R0049	false	510-MethyleneTHF + Gly = L-Ser + THF	serinehydroxymethyltransferase [2.1.2.1]
RN:R0050	false	L-HomoCys = L-Met	5-methyltetrahydropteroyltriglutamate-homocysteinemethyltransferase [2.1.1.14]

RN:R0052	false	5-O-(1-Carboxyvinyl)-3-PShi = Chor	chorismatesynthase [4.2.3.5]
RN:R0057	false	Anthranilate + PRPP = (5-P-D-R)anthranilate	anthranilatephosphoribosyltransferase [2.4.2.18]
RN:R0058	false	L-Asp + L-Gln = L-Asn + L-Glu	asparagine synthase [6.3.5.4]
RN:R0059	false	L-Asp = 4-P-L-Asp	asparto kinase II [2.7.2.4]
RN:R0061	false	L-Glu + NH3 = L-Gln	glutamine-ammonialigase [6.3.1.2]
RN:R0062	false	L-HomoSer = O-P-L-homoSer	homoserinekinase [2.7.1.39]
RN:R0065	false	Shi = Shi_3-P	shikimatekinase(SK) [2.7.1.71]
RN:R0068	false	Carbamoyl_P + L-Orn = L-Citrulline	ornithinecarbamoyltransferase [2.1.3.3]
RN:R0072	false	Chor + NH3 = Anthranilate + PYR	anthranilatesynthase [4.1.3.27]
RN:R0073	false	Chor = Prephenate	chorismatemutase [5.4.99.5]
RN:R0075	false	D-erythro-1-(Imidazol-4-yl)glycerol_3-P = 3-(Imidazol-4-yl)-2-oxopropyl_P	imidazoleglycerol-phosphatedehydratase [4.2.1.19]
RN:R0089	false	L-Arg = L-Citrulline + NH3	argininedeiminase [3.5.3.6]
RN:R0090	false	L-Arg = L-Orn + Urea	arginase [3.5.3.1]
RN:R0093	false	L-Glu + OAA = AKG + L-Asp	aspartate transaminase [2.6.1.1]
RN:R0095	false	L-Asp_4-semialdehyde + PYR = 23-Dihydrodipicolinate	dihydrodipicolinate synthase [4.2.1.52]
RN:R0100	true	L-Glu = D-Glu	glutamateracemase [5.1.1.3]
RN:R0104	false	L-Histidinol + 2 NAD+ = L-His + 2 NADH	putative histidinoldehydrogenase [1.1.1.23]
RN:R0105	false	L-Histidinol_P = L-Histidinol	putative histidinol phosphatase [3.1.3.15]
RN:R0106	true	L-HomoSer + NADP+ = L-Asp_4-semialdehyde + NADPH	homoserine dehydrogenase [1.1.1.3]
RN:R0112	false	L-Orn = L-Pro + NH3	putative ornithinecyclodeaminase [4.3.1.12]
RN:R0114	false	Ac-CoA + L-Ser = CoA + O-Ac-L-Ser	serineO-acetyltransferase [2.3.1.30]
RN:R0116	false	Indoleglycerol_P + L-Ser = GA_P + L-Try	tryptophan synthase [4.2.1.20]
RN:R0117	false	NH3 + PYR = L-Ser	thereoninedehydratase [4.3.1.19, 4.3.1.17]
RN:R0120	true	L-Thr = Acetaldehyde + Gly	putative threoninealdolase [4.1.2.5]
RN:R0121	false	L-Thr = 2-Oxobutanoate + NH3	thereoninedehydratase [4.3.1.19]
RN:R0125	false	Pribulosyl-formimino-AICAR-P = AICAR + D-erythro-1-(Imidazol-4-yl)glycerol_3-P	amidotransferasehisH [2.4.2.-]
RN:R0126	false	(5-P-D-R)anthranilate = 1-(2-Carboxyphenylamino)-1-deoxy-D-Ru_5-P	phosphoriborylanthranilateisomerase [5.3.1.24]
RN:R0134	false	Formimino-L-Glu = Formamide + L-Glu	hypothetical protein,similartoformiminoglutamase [3.5.3.8]
RN:R0138	false	O-P-L-homoSer = L-Thr	threoninesynthase [4.2.3.1]
RN:R0141	false	D-E4P + PEP = DAHP	phospho-2-dehydro-3-deoxyheptonate aldo [2.5.1.54]
RN:R0142	false	PEP + Shi_3-P = 5-O-(1-Carboxyvinyl)-3-PShi	3-phosphoshikimate1-carboxyvinyltransferase [2.5.1.19]
RN:R0143	false	PR-AMP = PR-formimino-AICAR-P	histidinebiosynthesisbifunctionalproteinHisIE [3.5.4.19]
RN:R0144	false	PR-ATP = PR-AMP	histidinebiosynthesisbifunctionalproteinHisIE [3.6.1.31]
RN:R0145	false	PRPP = PR-ATP	ATPphosphoribosyltransferase [2.4.2.17]
RN:R0146	false	NAD+ + Prephenate = 3-(4-Hydroxyphenyl)PYR + NADH	prephenatedehydrogenase [1.3.1.12]
RN:R0147	false	Prephenate = PhenylPYR	prephenate dehydratase [4.2.1.51]

RN:R0155	false	3-DehydroShi + NADPH = NADP+ + Shi	shikimatedehydrogenease [1.1.1.25]
RN:R0160	false	Urea = 2 NH3	urease [3.5.1.5]
RN:R0161	false	Urocanate = 4-Imidazolone-5-propanoate	urocanatehydratase [4.2.1.49]
RN:R0166	false	3-(Imidazol-4-yl)-2-oxopropyl_P + L-Glu = AKG + L-Histidinol_P	putative histidinol-phosphateaminotransferase [2.6.1.9]
RN:R0167	false	L-Glu + PhenylPYR = AKG + L-PhenylAla	putativehistidinol-phosphateaminotransferase [2.6.1.9]
RN:R0168	false	3-(4-Hydroxyphenyl)PYR + L-Glu = AKG + L-Tyr	putative histidinol-phosphatetransaminase [2.6.1.9]
RN:R0179	false	Acetaldehyde + NAD+ = Ac + NADH	aldehyde dehydrogenase [1.2.1.3]
RN:R0188	false	DHQ = 3-DehydroShi	enolase [4.2.1.11, 4.2.1.10]
RN:R0195	false	PYR + Thiamin_diP = 2-(A-Hydroxyethyl)thiamine_diP	pyruvate dehydrogenase_acetolactatesynthase [1.2.4.1, 2.2.1.6]
RN:R0198	false	O-Ac-L-Ser = Ac + L-Cys	cysteinesynthase [2.5.1.47]
RN:R0208	false	NH3 = Carbamoyl_P	carbamatekinase [2.7.2.2]
RN:R0209	false	D-Ala = L-Ala	alanineracemase [5.1.1.1]
RN:R0238	false	GA_P + NAD+ = 3-P-D-glyceroyl_P + NADH	glyceraldehyde-3-phosphate dehydrogenase [1.2.1.12]
RN:R0239	false	Glycerone_P = GA_P	triosephosphate isomerase [5.3.1.1]
RN:R0243	false	Mal + Menaquinone = Menaquinol + OAA	malate:quinoneoxidoreductase [1.1.99.16]
RN:R0245	true	Mal + NAD+ = NADH + PYR	malatedehydrogenase homolog [1.1.1.38]
RN:R0246	false	Fum = Mal	fumarate hydratase, class-II [4.2.1.2]
RN:R0255	false	3-P-D-GA = 2-P-D-GA	phosphoglycerate mutase [5.4.2.1]
RN:R0256	false	2-P-D-GA = PEP	enolase [4.2.1.11]
RN:R0260	false	6PGlucon + NADP+ = D-Ru_5-P + NADPH	phosphogluconate dehydrogenase [1.1.1.44]
RN:R0267	false	Ac-CoA + OAA = Cit + CoA	citrate synthase [2.3.3.1]
RN:R0269	false	PEP = PYR	pyruvate kinase [2.7.1.40]
RN:R0270	false	3-P-D-glyceroyl_P = 3-P-D-GA	phosphoglycerate kinase [2.7.2.3]
RN:R0271	false	Ac + CoA = Ac-CoA	acetyl-CoA synthetase AND ligase [6.2.1.1]
RN:R0274	false	D-F = D-F_6-P	fructokinase,putative [2.7.1.4]
RN:R0276	false	D-F_6-P = D-F_16-BP	6-phosphofructokinase [2.7.1.11]
RN:R0280	false	D-Ri_5-P = PRPP	ribose-phosphate pyrophosphokinase [2.7.6.1]
RN:R0284	false	OAA = PEP	phosphoenolpyruvate carboxykinase [4.1.1.49]
RN:R0287	false	PYR = OAA	pyruvate carboxylase [6.4.1.1]
RN:R0288	true	CoA + Succ = SuccCoA	succinyl-CoA synthetase [6.2.1.5]
RN:R0294	false	D-F_16-BP = D-F_6-P	fructose-bisphosphatase [3.1.3.11]
RN:R0295	true	D-F_6-P + GA_P = D-E4P + D-Xu_5-P	transketolase [2.2.1.1]
RN:R0297	false	D-Glucono-15-lactone_6-P = 6PGlucon	Spon_PGLactonohydrolase [3.1.1.31]
RN:R0301	false	D-G_6-P + NADP+ = D-Glucono-15-lactone_6-P + NADPH	glucose-6-phosphate 1-dehydrogenase [1.1.1.49]
RN:R0302	false	D-G_6-P = D-F_6-P	glucose-6-phosphate isomerase A [5.3.1.9]
RN:R0311	false	D-Ru_5-P = D-Ri_5-P	ribose 5-phosphate isomerase A [5.3.1.6]
RN:R0314	false	D-Ru_5-P = D-Xu_5-P	ribulose-phosphate 3-epimerase [5.1.3.1]
RN:R0315	true	D-S7P + GA_P = D-E4P + D-F_6-P	Transaldolase superfamily [2.2.1.2]

RN:R0316	true	D-S7P + GA_P = D-Ri_5-P + D-Xu_5-P	transketolase [2.2.1.1]
RN:R0324	false	IsoCit + NADP+ = NADPH + OASucc	isocitrate dehyrogenase [1.1.1.42]
RN:R0335	false	OASucc = AKG	isocitrate dehyrogenase [1.1.1.42]
RN:R0345	false	FAD + Succ = FADH2 + Fum	succinate dehydrogenase [1.3.99.1]
RN:R0348	false	Sucrose_6-P = D-F + D-G_6-P	sucrose-6-phosphatehydrolase [3.2.1.26]
RN:R0358	false	cis-Acon = IsoCit	aconitate hydratase [4.2.1.3]
RN:R0359	false	Cit = cis-Acon	aconitate hydratase [4.2.1.3]
RN:R0360	false	D-F_16-BP = GA_P + Glycerone_P	fructose-bisphosphate aldolase [4.1.2.13]
RN:R0372	false	AKG + L-Gln + NADPH = 2 L-Glu + NADP+	glutamatesynthase [1.4.1.13]
RN:R0384	false	L-His = NH3 + Urocanate	histidineammonia-lyase [4.3.1.3]
RN:R0522	true	NH3(ex) = NH3	probabale ammonium transporter [2.A.49]
RN:R0534	false	D-G(ex) + PEP = D-G_6-P + PYR	glucose-specific PTS [2.7.1.69, 2.7.3.9]
RN:R0673	true	AKG + D-Ala = D-Glu + PYR	D-alanineaminotransferase [2.6.1.21]
RN:R0986	false	2 L-Glu + NADH = AKG + L-Orn + NAD+	EZComp_UreaOrnSyn [1.5.1.12, 2.6.1.13]
RN:R0987	false	2345-Tetrahydrodipicolinate + L-Glu + SuccCoA = AKG + CoA + L-Lys + Succ	EZComp_LysSyn [2.3.1.117, 2.6.1.17, 3.5.1.18, 5.1.1.7, 4.1.1.20]
RN:R0988	false	AKG + CoA + NAD+ = NADH + SuccCoA	EZComp_TCASuccinyl [1.2.4.2,2.3.1.61, 1.8.1.4]
RN:R0989	false	2-(A-Hydroxyethyl)thiamine_diP + 2-Oxobutanoate + L-Glu + NADPH = AKG + L-Ile + NADP+ + Thiamin_diP	EZComp_Ile_Meta [2.2.1.6, 1.1.1.86, 4.2.1.9, 2.6.1.42]
RN:R0990	false	2-(A-Hydroxyethyl)thiamine_diP + NADPH + PYR = 3-Methyl-2-oxobutanoate + NADP+ + Thiamin_diP	EZComp_Val_Meta [2.2.1.6, 1.1.1.86, 4.2.1.9]
RN:R0991	false	3-Methyl-2-oxobutanoate + Ac-CoA + L-Glu + NAD+ = AKG + CoA + L-Leu + NADH	EZComp_Leu_Meta [2.3.3.13, 4.2.1.33, 1.1.1.85, 2.6.1.42]
RN:R0993	false	L-Asp + L-Citrulline = Fum + L-Arg	EZComp_Fum_Asp [4.3.2.1, 6.3.4.5]
RN:R0994	false	CoA + NAD+ + PYR = Ac-CoA + NADH	EZComp_PyrAcetylCoA [1.2.4.1, 2.3.1.12, 1.8.1.4]
RN:R0995	false	Gly + NAD+ + THF = 510-MethyleneTHF + NADH + NH3	EZComp_GlySerThrMeta [2.1.2.10, 1.8.1.4, 1.4.4.2]
RN:R0996	false	Ac-CoA + L-Cys + L-HomoSer = Ac + CoA + L-HomoCys + NH3 + PYR	EZComp_MetMeta [2.3.1.31, 2.5.1.48, 4.4.1.8]
RN:R0997	false	AKG + L-Orn + NADH + 2 NADPH = 2 L-Pro + NAD+ + 2 NADP+	EZComp_UreaMeta [1.5.1.2, 1.5.1.12, 2.6.1.13]

Table 12: *S. aureus* network - Elementary Modes discussed in the results section[1]

#	Flux sum	Rev.?[2]	Length	Reactions[3]	Net reaction
3	25	false	16	(1 RN:Nucl02) (2 RN:R0061) (2 RN:R0089) (2 RN:R0093) (2 RN:R0245) (2 RN:R0246) (1 RN:R0260) (1 RN:R0280) (1 RN:R0284) (3 RN:R0287) (1 RN:R0297) (1 RN:R0301) (1 RN:R0311) (2 RN:R0372) (1 RN:R0534) (2 RN:R0993)	D-G(ex) + 2 NAD+ = 2 NADH + Pyrimidine(ex)
19	428	false	35	(5 RN:E0001) (11 RN:E0019) (5 RN:R0021) (11 RN:R0038) (5 RN:R0052) (16 RN:R0061) (5 RN:R0065) (5 RN:R0073) (5 RN:R0141) (5 RN:R0142) (5 RN:R0147) (5 RN:R0155) (5 RN:R0167) (5 RN:R0188) (11 RN:R0195) (32 RN:R0238) (15 RN:R0239) (32 RN:R0255) (32 RN:R0256) (16 RN:R0260) (32 RN:R0270) (15 RN:R0276) (-2 RN:R0295) (16 RN:R0297) (16 RN:R0301) (6 RN:R0302) (7 RN:R0311) (9 RN:R0314) (7 RN:R0315) (-7 RN:R0316) (15 RN:R0360) (16 RN:R0372) (16 RN:R0522) (22 RN:R0534) (11 RN:R0990)	22 D-G(ex) + 32 NAD+ + 16 NH3(ex) = 32 NADH + 5 Phe-tRNA(ex) + 11 Val-tRNA(ex)
21	239	false	36	(3 RN:E0001) (11 RN:E0009) (3 RN:R0021) (3 RN:R0052) (3 RN:R0061) (3 RN:R0065) (3 RN:R0073) (11 RN:R0114) (11 RN:R0117) (3 RN:R0141) (3 RN:R0142) (3 RN:R0147) (3 RN:R0155) (3 RN:R0167) (3 RN:R0188) (11 RN:R0198) (17 RN:R0238) (9 RN:R0239) (17 RN:R0255) (17 RN:R0256) (3 RN:R0260) (17 RN:R0270) (11 RN:R0271) (9 RN:R0276) (1 RN:R0295) (3 RN:R0297) (3 RN:R0301) (8 RN:R0302) (2 RN:R0311) (1 RN:R0314) (2 RN:R0315) (-2 RN:R0316) (9 RN:R0360) (3 RN:R0372) (14 RN:R0522) (11 RN:R0534)	11 D-G(ex) + 17 NAD+ + 14 NH3(ex) = 11 Cys-tRNA(ex) + 17 NADH + 3 Phe-tRNA(ex)
81	289	false	36	(5 RN:E0013) (5 RN:E0018) (6 RN:Nucl02) (5 RN:R0047) (5 RN:R0059) (15 RN:R0061) (5 RN:R0062) (5 RN:R0093) (5 RN:R0100) (-5 RN:R0106) (5 RN:R0121) (5 RN:R0138) (5 RN:R0195) (5 RN:R0209) (15 RN:R0238) (6 RN:R0239) (15 RN:R0255) (15 RN:R0256) (15 RN:R0260) (15 RN:R0270) (6 RN:R0276) (6 RN:R0280) (5 RN:R0287) (-3 RN:R0295) (15 RN:R0297) (15 RN:R0301) (9 RN:R0311) (6 RN:R0314) (3 RN:R0315) (-3 RN:R0316) (6 RN:R0360) (15 RN:R0372) (10 RN:R0522) (15 RN:R0534) (-5 RN:R0673) (5 RN:R0989)	15 D-G(ex) + 15 NAD+ + 10 NH3(ex) = 5 Ala-tRNA(ex) + 5 Ile-tRNA(ex) + 15 NADH + 6 Pyrimidine(ex)

91	853	false	42	(13 RN:E0001) (10 RN:E0018) (13 RN:R0021) (10 RN:R0047) (13 RN:R0052) (10 RN:R0059) (33 RN:R0061) (10 RN:R0062) (13 RN:R0065) (13 RN:R0073) (10 RN:R0093) (-10 RN:R0106) (10 RN:R0121) (10 RN:R0138) (13 RN:R0141) (13 RN:R0142) (13 RN:R0147) (13 RN:R0155) (13 RN:R0167) (13 RN:R0188) (10 RN:R0195) (46 RN:R0238) (21 RN:R0239) (46 RN:R0255) (46 RN:R0256) (38 RN:R0260) (46 RN:R0270) (21 RN:R0276) (18 RN:R0284) (28 RN:R0287) (-4 RN:R0295) (38 RN:R0297) (38 RN:R0301) (17 RN:R0311) (21 RN:R0314) (17 RN:R0315) (-17 RN:R0316) (21 RN:R0360) (33 RN:R0372) (23 RN:R0522) (38 RN:R0534) (10 RN:R0989)	38 D-G(ex) + 46 NAD+ + 23 NH3(ex) = 10 Ile-tRNA(ex) + 46 NADH + 13 Phe-tRNA(ex)
100	241	false	40	(3 RN:E0001) (2 RN:E0006) (10 RN:E0008) (3 RN:R0021) (2 RN:R0047) (3 RN:R0052) (2 RN:R0059) (5 RN:R0061) (2 RN:R0062) (3 RN:R0065) (3 RN:R0073) (2 RN:R0093) (-2 RN:R0106) (10 RN:R0117) (2 RN:R0138) (3 RN:R0141) (3 RN:R0142) (3 RN:R0147) (3 RN:R0155) (3 RN:R0167) (3 RN:R0188) (18 RN:R0238) (9 RN:R0239) (18 RN:R0255) (18 RN:R0256) (6 RN:R0260) (18 RN:R0270) (9 RN:R0276) (2 RN:R0287) (6 RN:R0297) (6 RN:R0301) (6 RN:R0302) (3 RN:R0311) (3 RN:R0314) (3 RN:R0315) (-3 RN:R0316) (9 RN:R0360) (5 RN:R0372) (15 RN:R0522) (12 RN:R0534)	12 D-G(ex) + 18 NAD+ + 15 NH3(ex) = 18 NADH + 3 Phe-tRNA(ex) + 10 Ser-tRNA(ex) + 2 Thr-tRNA(ex)

[1] *complete list availabe from the authors on request*

[2] *reversible*

[3] *reaction identifiers are taken from KEGG, for annotations see the reaction table*

Table 13: *S. epidermidis* network - metabolites

Name	internal?	Connectivity	Description
(5-P-D-R)anthranilate	true	2	N-(5-Phospho-D-ribosyl)anthranilate
1-(2-Carboxyphenylamino)-1-deoxy-D-Ru_5-P	true	2	1-(2-Carboxyphenylamino)-1-deoxy-D-ribulose_5-phosphate
2-(A-Hydroxyethyl)thiamine_diP	true	3	2-(alpha-Hydroxyethyl)thiamine_diphosphate
2-Oxobutanoate	true	2	2-Oxobutanoate
2-P-D-GA	true	2	2-Phospho-D-glycerate
23-Dihydrodipicolinate	true	2	2,3-Dihydrodipicolinate
2345-Tetrahydrodipicolinate	true	2	2,3,4,5-Tetrahydrodipicolinate
3-(4-Hydroxyphenyl)PYR	true	2	3-(4-Hydroxyphenyl)pyruvate
3-(Imidazol-4-yl)-2-oxopropyl_P	true	2	3-(Imidazol-4-yl)-2-oxopropyl_phosphate
3-DehydroShi	true	2	3-Dehydroshikimate
3-Methyl-2-oxobutanoate	true	3	3-Methyl-2-oxobutanoate
3-P-D-GA	true	2	3-Phospho-D-glycerate
3-P-D-glyceroyl_P	true	2	3-Phospho-D-glyceroyl_phosphate
4-Imidazolone-5-propanoate	true	1	4-Imidazolone-5-propanoate
4-P-L-Asp	true	2	4-Phospho-L-aspartate
5-O-(1-Carboxyvinyl)-3-PShi	true	2	5-O-(1-Carboxyvinyl)-3-phosphoshikimate
510-MethyleneTHF	true	2	5,10-Methylenetetrahydrofolate
6PGlucon	true	2	6-Phospho-D-gluconate
Ac	true	4	Acetate
Ac-CoA	true	6	Acetyl-CoA
Acetaldehyde	true	2	Acetaldehyde
AICAR	true	2	1-(5-Phosphoribosyl)-5-amino-4-imidazolecarboxamide
AKG	true	15	2-Oxoglutarate
Ala-tRNA(ex)	false	1	Ala-tRNA(ex)
Anthranilate	true	2	Anthranilate
Arg-tRNA(ex)	false	1	Arg-tRNA(ex)
Asn-tRNA(ex)	false	1	Asn-tRNA(ex)
Asp-tRNA(ex)	false	1	Asp-tRNA(ex)
Carbamoyl_P	true	3	Carbamoyl_phosphate
Chor	true	3	Chorismate
cis-Acon	true	2	cis-Aconitate
Cit	true	2	Citrate
CoA	true	9	CoA
Cys-tRNA(ex)	false	1	Cys-tRNA(ex)
D-Ala	true	2	D-Alanine
D-E4P	true	3	D-Erythrose_4-phosphate
D-erythro-1-(Imidazol-4-yl)glycerol_3-P	true	2	D-erythro-1-(Imidazol-4-yl)glycerol_3-phosphate

D-F	true	2	D-Fructose
D-F_16-BP	true	3	D-Fructose_1,6-bisphosphate
D-F_6-P	true	6	D-Fructose_6-phosphate
D-G(ex)	false	1	D-Glucose(ex)
D-G_6-P	true	4	D-Glucose_6-phosphate
D-Glu	true	2	D-Glutamate
D-Glucono-15-lactone_6-P	true	2	D-Glucono-1,5-lactone_6-phosphate
D-Ri_5-P	true	3	D-Ribose_5-phosphate
D-Ru_5-P	true	3	D-Ribulose_5-phosphate
D-S7P	true	2	D-Sedoheptulose_7-phosphate
D-Xu_5-P	true	3	D-Xylulose_5-phosphate
DAHP	true	2	2-Dehydro-3-deoxy-D-arabino-heptonate_7-phosphate
DHQ	true	2	3-Dehydroquinate
FAD(ex)	false	1	FAD
FADH2(ex)	false	1	FADH2
Formamide	true	1	Formamide
Formimino-L-Glu	true	2	N-Formimino-L-glutamate
Fum	true	3	Fumarate
GA_P	true	7	(2R)-2-Hydroxy-3-(phosphonooxy)-propanal
Gln-tRNA(ex)	false	1	Gln-tRNA(ex)
Glu-tRNA(ex)	false	1	Glu-tRNA(ex)
Gly	true	4	Glycine
Gly-tRNA(ex)	false	1	Gly-tRNA(ex)
Glycerone_P	true	2	Glycerone_phosphate
His-tRNA(ex)	false	1	His-tRNA(ex)
Ile-tRNA(ex)	false	1	Ile-tRNA(ex)
Indoleglycerol_P	true	2	Indoleglycerol_phosphate
IsoCit	true	2	Isocitrate
L-Ala	true	2	L-Alanine
L-Arg	true	3	L-Arginine
L-Asn	true	2	L-Asparagine
L-Asp	true	5	L-Aspartate
L-Asp_4-semialdehyde	true	3	L-Aspartate_4-semialdehyde
L-Citrulline	true	3	L-Citrulline
L-Cys	true	3	L-Cysteine
L-Gln	true	5	L-Glutamine
L-Glu	true	18	L-Glutamate
L-Glu_5-semialdehyde	true	2	L-Glutamate_5-semialdehyde
L-His	true	2	L-Histidine
L-Histidinol	true	2	L-Histidinol

L-Histidinol_P	true	2	L-Histidinol_phosphate
L-HomoCys	true	2	L-Homocysteine
L-HomoSer	true	3	L-Homoserine
L-Ile	true	2	L-Isoleucine
L-Leu	true	2	L-Leucine
L-Lys	true	2	L-Lysine
L-Met	true	2	L-Methionine
L-Orn	true	5	L-Ornithine
L-PhenylAla	true	2	L-Phenylalanine
L-Pro	true	3	L-Proline
L-Ser	true	5	L-Serine
L-Thr	true	4	L-Threonine
L-Try	true	2	L-Tryptophane
L-Tyr	true	2	L-Tyrosine
L-Val	true	2	L-Valine
Leu-tRNA(ex)	false	1	Leu-tRNA(ex)
Lys-tRNA(ex)	false	1	Lys-tRNA(ex)
Mal	true	4	(S)-Malate
Menaquinol	true	1	Menaquinol
Menaquinone	true	1	Menaquinone
Met-tRNA(ex)	false	1	Met-tRNA(ex)
NAD+	false	13	NAD+
NADH	false	13	NADH
NADP+	true	11	NADP+
NADPH	true	11	NADPH
NH3	true	11	NH3
NH3(ex)	false	1	NH3(ex)
O-Ac-L-Ser	true	2	O-Acetyl-L-serine
O-P-L-homoSer	true	2	O-Phospho-L-homoserine
OAA	true	6	Oxaloacetate
OASucc	true	2	Oxalosuccinate
PEP	true	6	Phosphoenolpyruvate
Phe-tRNA(ex)	false	1	Phe-tRNA(ex)
PhenylPYR	true	2	Phenylpyruvate
PR-AMP	true	2	Phosphoribosyl-AMP
PR-ATP	true	2	Phosphoribosyl-ATP
PR-formimino-AICAR-P	true	2	5-(5-Phospho-D-ribosylaminoformimino)-1-(5-phosphoribosyl)-imidazole-4-carboxamide
Prephenate	true	3	Prephenate
Pribulosyl-formimino-AICAR-P	true	2	N-(5-Phospho-D-1-ribulosylformimino)-5-amino-1-(5-phospho-D-ribosyl)-4-imidazolecarboxamide
Pro-tRNA(ex)	false	1	Pro-tRNA(ex)

PRPP	true	4	5-Phospho-D-ribose_1-diphosphate
Purine(ex)	false	1	Purine(ex)
PYR	true	12	Pyruvate
Pyrimidine(ex)	false	1	Pyrimidine(ex)
Ser-tRNA(ex)	false	1	Ser-tRNA(ex)
Shi	true	2	Shikimate
Shi_3-P	true	2	Shikimate_3-phosphate
Succ	true	3	Succinate
SuccCoA	true	3	Succinyl-CoA
Sucrose_6-P	true	1	Sucrose_6-phosphate
THF	true	2	Tetrahydrofolate
Thiamin_diP	true	3	Thiamin_diphosphate
Thr-tRNA(ex)	false	1	Thr-tRNA(ex)
Try-tRNA(ex)	false	1	Try-tRNA(ex)
Tyr-tRNA(ex)	false	1	Tyr-tRNA(ex)
Urea	true	1	Urea
Val-tRNA(ex)	false	1	Val-tRNA(ex)

Table 14: *S. epidermidis* network - reactions

Enzyme name	rev.?	Reaction equation	Annotation
rn:E0001	false	L-PhenylAla = Phe-tRNA(ex)	PheX [6.1.1.20]
rn:E0002	false	L-Tyr = Tyr-tRNA(ex)	TyrX [6.1.1.1]
rn:E0003	false	L-Try = Try-tRNA(ex)	TryX [6.1.1.-]
rn:E0004	false	L-His = His-tRNA(ex)	HisX [6.1.1.21]
rn:E0005	false	L-Met = Met-tRNA(ex)	MetX [6.1.1.10]
rn:E0006	false	L-Thr = Thr-tRNA(ex)	ThrX [6.1.1.3]
rn:E0007	false	Gly = Gly-tRNA(ex)	GlyX [6.1.1.14]
rn:E0008	false	L-Ser = Ser-tRNA(ex)	SerX [6.1.1.11]
rn:E0009	false	L-Cys = Cys-tRNA(ex)	CysX [6.1.1.16]
rn:E0010	false	L-Lys = Lys-tRNA(ex)	LysX [6.1.1.6]
rn:E0011	false	L-Asn = Asn-tRNA(ex)	AsnX [6.1.1.22]
rn:E0012	false	L-Asp = Asp-tRNA(ex)	AspX [6.1.1.12]
rn:E0013	false	L-Ala = Ala-tRNA(ex)	AlaX [6.1.1.7]
rn:E0014	false	L-Pro = Pro-tRNA(ex)	ProX [6.1.1.15]
rn:E0015	false	L-Arg = Arg-tRNA(ex)	ArgX [6.1.1.19]
rn:E0016	false	L-Glu = Glu-tRNA(ex)	GluX [6.1.1.17]
rn:E0017	false	L-Gln = Gln-tRNA(ex)	GlnX [6.1.1.17]
rn:E0018	false	L-Ile = Ile-tRNA(ex)	IleX [6.1.1.5]
rn:E0019	false	L-Val = Val-tRNA(ex)	ValX [6.1.1.9]
rn:E0020	false	L-Leu = Leu-tRNA(ex)	LeuX [6.1.1.4]
rn:N01	false	AICAR = Purine(ex)	PurineMeta []
rn:N02	false	PRPP = Pyrimidine(ex)	PyrimidineMeta []
rn:U0015	false	1-(2-Carboxyphenylamino)-1-deoxy-D-Ru_5-P = Indoleglycerol_P	indole-3-glycerolphosphatesynthase [4.1.1.48]
rn:U0018	false	L-Gln = Carbamoyl_P + L-Glu	carbamoyl-phosphatesynthase [6.3.5.5]
rn:U0020	false	23-Dihydrodipicolinate + NADPH = 2345-Tetrahydrodipicolinate + NADP+	dihydrodipicolinate reductase [1.3.1.26]
rn:U0021	false	DAHP = DHQ	3-dehydroquinatesynthase [4.2.3.4]
rn:U0038	false	3-Methyl-2-oxobutanoate + L-Glu = AKG + L-Val	branched-chainaminoacidaminotransferase [2.6.1.42]
rn:U0045	false	4-Imidazolone-5-propanoate = Formimino-L-Glu	imidazolonepropionase [3.5.2.7]
rn:U0047	false	4-P-L-Asp + NADPH = L-Asp_4-semialdehyde + NADP+	aspartate semialdehyde dehydrogenase [1.2.1.11]
rn:U0048	false	PR-formimino-AICAR-P = Pribulosyl-formimino-AICAR-P	putative phosphoribosylformimino-5-aminoimidazolecarboxamideribotidei-somerase [5.3.1.16]
rn:U0049	false	510-MethyleneTHF + Gly = L-Ser + THF	serinehydroxymethyltransferase [2.1.2.1]
rn:U0050	false	L-HomoCys = L-Met	5-methyltetrahydropteroyltriglutamate-homocysteinemethyltransferase [2.1.1.14]

rn:U0052	false	5-O-(1-Carboxyvinyl)-3-PShi = Chor	chorismatesynthase [4.2.3.5]
rn:U0057	false	Anthranilate + PRPP = (5-P-D-R)anthranilate	anthranilatephosphoribosyltransferase [2.4.2.18]
rn:U0058	false	L-Asp + L-Gln = L-Asn + L-Glu	asparagine synthase [6.3.5.4]
rn:U0059	false	L-Asp = 4-P-L-Asp	asparto kinase II [2.7.2.4]
rn:U0061	false	L-Glu + NH3 = L-Gln	glutamine-ammonialigase [6.3.1.2]
rn:U0062	false	L-HomoSer = O-P-L-homoSer	homoserinekinase [2.7.1.39]
rn:U0065	false	Shi = Shi_3-P	shikimatekinase(SK) [2.7.1.71]
rn:U0068	false	Carbamoyl_P + L-Orn = L-Citrulline	ornithinecarbamoyltransferase [2.1.3.3]
rn:U0072	false	Chor + NH3 = Anthranilate + PYR	anthranilatesynthase [4.1.3.27]
rn:U0073	false	Chor = Prephenate	chorismatemutase [5.4.99.5]
rn:U0075	false	D-erythro-1-(Imidazol-4-yl)glycerol_3-P = 3-(Imidazol-4-yl)-2-oxop-ropyl_P	imidazoleglycerol-phosphatedehydratase [4.2.1.19]
rn:U0089	false	L-Arg = L-Citrulline + NH3	argininedeiminase [3.5.3.6]
rn:U0093	false	L-Glu + OAA = AKG + L-Asp	aspartate transaminase [2.6.1.1]
rn:U0095	false	L-Asp_4-semialdehyde + PYR = 23-Dihydrodipicolinate	dihydrodipicolinate synthase [4.2.1.52]
rn:U0100	true	L-Glu = D-Glu	glutamateracemase [5.1.1.3]
rn:U0101	true	L-Glu_5-semialdehyde + NAD+ = L-Glu + NADH	1-pyrroline-5-carboxylatedehydrogenase [1.5.1.12]
rn:U0104	false	L-Histidinol + 2 NAD+ = L-His + 2 NADH	putative histidinoldehydrogenase [1.1.1.23]
rn:U0105	false	L-Histidinol_P = L-Histidinol	putative histidinol phosphatase [3.1.3.15]
rn:U0106	true	L-HomoSer + NADP+ = L-Asp_4-semialdehyde + NADPH	homoserine dehydrogenase [1.1.1.3]
rn:U0111	true	AKG + L-Orn = L-Glu + L-Glu_5-semialdehyde	ornithineaminotransferase [2.6.1.13]
rn:U0112	false	L-Orn = L-Pro + NH3	putative ornithinecyclodeaminase [4.3.1.12]
rn:U0114	false	Ac-CoA + L-Ser = CoA + O-Ac-L-Ser	serineO-acetyltransferase [2.3.1.30]
rn:U0116	false	Indoleglycerol_P + L-Ser = GA_P + L-Try	tryptophan synthase [4.2.1.20]
rn:U0117	false	NH3 + PYR = L-Ser	thereoninedehydratase [4.3.1.19, 4.3.1.17]
rn:U0120	true	L-Thr = Acetaldehyde + Gly	putative threoninealdolase [4.1.2.5]
rn:U0121	false	L-Thr = 2-Oxobutanoate + NH3	thereoninedehydratase [4.3.1.19]
rn:U0125	false	Pribulosyl-formimino-AICAR-P = AICAR + D-erythro-1-(Imidazol-4-yl)glycerol_3-P	amidotransferasehisH [2.4.2.-]
rn:U0126	false	(5-P-D-R)anthranilate = 1-(2-Carboxyphenylamino)-1-deoxy-D-Ru_5-P	phosphoriborylanthranilateisomerase [5.3.1.24]
rn:U0134	false	Formimino-L-Glu = Formamide + L-Glu	hypothetical protein,similartoformiminoglutamase [3.5.3.8]
rn:U0138	false	O-P-L-homoSer = L-Thr	threoninesynthase [4.2.3.1]
rn:U0141	false	D-E4P + PEP = DAHP	phospho-2-dehydro-3-deoxyheptonate aldo [2.5.1.54]
rn:U0142	false	PEP + Shi_3-P = 5-O-(1-Carboxyvinyl)-3-PShi	3-phosphoshikimate1-carboxyvinyltransferase [2.5.1.19]
rn:U0143	false	PR-AMP = PR-formimino-AICAR-P	histidinebiosynthesisbifunctionalproteinHisIE [3.5.4.19]
rn:U0144	false	PR-ATP = PR-AMP	histidinebiosynthesisbifunctionalproteinHisIE [3.6.1.31]
rn:U0145	false	PRPP = PR-ATP	ATPphosphoribosyltransferase [2.4.2.17]

rn:U0146	false	NAD+ + Prephenate = 3-(4-Hydroxyphenyl)PYR + NADH	prephenatedehydrogenase [1.3.1.12]
rn:U0147	false	Prephenate = PhenylPYR	prephenate dehydratase [4.2.1.51]
rn:U0155	false	3-DehydroShi + NADPH = NADP+ + Shi	shikimatedehydrogenease [1.1.1.25]
rn:U0160	false	Urea = 2 NH3	urease [3.5.1.5]
rn:U0166	false	3-(Imidazol-4-yl)-2-oxopropyl_P + L-Glu = AKG + L-Histidinol_P	putative histidinol-phosphateaminotransferase [2.6.1.9]
rn:U0167	false	L-Glu + PhenylPYR = AKG + L-PhenylAla	putativehistidinol-phosphateaminotransferase [2.6.1.9]
rn:U0168	false	3-(4-Hydroxyphenyl)PYR + L-Glu = AKG + L-Tyr	putative histidinol-phosphatetransaminase [2.6.1.9]
rn:U0179	false	Acetaldehyde + NAD+ = Ac + NADH	aldehyde dehydrogenase [1.2.1.3]
rn:U0188	false	DHQ = 3-DehydroShi	enolase [4.2.1.11, 4.2.1.10]
rn:U0195	false	PYR + Thiamin_diP = 2-(A-Hydroxyethyl)thiamine_diP	pyruvate dehydrogenase_acetolactatesynthase [1.2.4.1, 2.2.1.6]
rn:U0198	false	O-Ac-L-Ser = Ac + L-Cys	cysteinesynthase [2.5.1.47]
rn:U0208	false	NH3 = Carbamoyl_P	carbamatekinase [2.7.2.2]
rn:U0209	false	D-Ala = L-Ala	alanineracemase [5.1.1.1]
rn:U0238	false	GA_P + NAD+ = 3-P-D-glyceroyl_P + NADH	glyceraldehyde-3-phosphate dehydrogenase [1.2.1.12]
rn:U0239	false	Glycerone_P = GA_P	triosephosphate isomerase [5.3.1.1]
rn:U0243	false	Mal + Menaquinone = Menaquinol + OAA	malate:quinoneoxidoreductase [1.1.99.16]
rn:U0244	false	Mal + NAD+ = NADH + OAA	MDH [1.1.1.37]
rn:U0245	true	Mal + NAD+ = NADH + PYR	malatedehydrogenase homolog [1.1.1.38]
rn:U0246	false	Fum = Mal	fumarate hydratase, class-II [4.2.1.2]
rn:U0255	false	3-P-D-GA = 2-P-D-GA	phosphoglycerate mutase [5.4.2.1]
rn:U0256	false	2-P-D-GA = PEP	enolase [4.2.1.11]
rn:U0260	false	6PGlucon + NADP+ = D-Ru_5-P + NADPH	phosphogluconate dehydrogenase [1.1.1.44]
rn:U0267	false	Ac-CoA + OAA = Cit + CoA	citrate synthase [2.3.3.1]
rn:U0269	false	PEP = PYR	pyruvate kinase [2.7.1.40]
rn:U0270	false	3-P-D-glyceroyl_P = 3-P-D-GA	phosphoglycerate kinase [2.7.2.3]
rn:U0271	false	Ac + CoA = Ac-CoA	acetyl-CoA synthetase AND ligase [6.2.1.1]
rn:U0274	false	D-F = D-F_6-P	fructokinase,putative [2.7.1.4]
rn:U0276	false	D-F_6-P = D-F_16-BP	6-phosphofructokinase [2.7.1.11]
rn:U0280	false	D-Ri_5-P = PRPP	ribose-phosphate pyrophosphokinase [2.7.6.1]
rn:U0284	false	OAA = PEP	phosphoenolpyruvate carboxykinase [4.1.1.49]
rn:U0287	false	PYR = OAA	pyruvate carboxylase [6.4.1.1]
rn:U0288	true	CoA + Succ = SuccCoA	succinyl-CoA synthetase [6.2.1.5]
rn:U0294	false	D-F_16-BP = D-F_6-P	fructose-bisphosphatase [3.1.3.11]
rn:U0295	true	D-F_6-P + GA_P = D-E4P + D-Xu_5-P	transketolase [2.2.1.1]
rn:U0297	false	D-Glucono-15-lactone_6-P = 6PGlucon	Spon_PGLactonohydrolase [3.1.1.31]
rn:U0301	false	D-G_6-P + NADP+ = D-Glucono-15-lactone_6-P + NADPH	glucose-6-phosphate 1-dehydrogenase [1.1.1.49]
rn:U0302	false	D-G_6-P = D-F_6-P	glucose-6-phosphate isomerase A [5.3.1.9]
rn:U0311	false	D-Ru_5-P = D-Ri_5-P	ribose 5-phosphate isomerase A [5.3.1.6]

rn:U0314	false	D-Ru_5-P = D-Xu_5-P	ribulose-phosphate 3-epimerase [5.1.3.1]
rn:U0315	true	D-S7P + GA_P = D-E4P + D-F_6-P	Transaldolase superfamily [2.2.1.2]
rn:U0316	true	D-S7P + GA_P = D-Ri_5-P + D-Xu_5-P	transketolase [2.2.1.1]
rn:U0324	false	IsoCit + NADP+ = NADPH + OASucc	isocitrate dehyrogenase [1.1.1.42]
rn:U0335	false	OASucc = AKG	isocitrate dehyrogenase [1.1.1.42]
rn:U0345	false	FAD(ex) + Succ = FADH2(ex) + Fum	succinate dehydrogenase [1.3.99.1]
rn:U0348	false	Sucrose_6-P = D-F + D-G_6-P	sucrose-6-phosphatehydrolase [3.2.1.26]
rn:U0358	false	cis-Acon = IsoCit	aconitate hydratase [4.2.1.3]
rn:U0359	false	Cit = cis-Acon	aconitate hydratase [4.2.1.3]
rn:U0360	false	D-F_16-BP = GA_P + Glycerone_P	fructose-bisphosphate aldolase [4.1.2.13]
rn:U0372	false	AKG + L-Gln + NADPH = 2 L-Glu + NADP+	glutamatesynthase [1.4.1.13]
rn:U0522	true	NH3(ex) = NH3	probabale ammonium transporter [2.A.49]
rn:U0534	false	D-G(ex) + PEP = D-G_6-P + PYR	glucose-specific PTS [2.7.1.69, 2.7.3.9]
rn:U0673	true	AKG + D-Ala = D-Glu + PYR	D-alanineaminotransferase [2.6.1.21]
rn:U0986	false	2 L-Glu + NADH = AKG + L-Orn + NAD+	EZComp_UreaOrnSyn [1.5.1.12, 2.6.1.13]
rn:U0987	false	2345-Tetrahydrodipicolinate + L-Glu + SuccCoA = AKG + CoA + L-Lys + Succ	EZComp_LysSyn [2.3.1.117, 2.6.1.17, 3.5.1.18, 5.1.1.7, 4.1.1.20]
rn:U0988	false	AKG + CoA + NAD+ = NADH + SuccCoA	EZComp_TCASuccinyl [1.2.4.2,2.3.1.61, 1.8.1.4]
rn:U0989	false	2-(A-Hydroxyethyl)thiamine_diP + 2-Oxobutanoate + L-Glu + NADPH = AKG + L-Ile + NADP+ + Thiamin_diP	EZComp_Ile_Meta [2.2.1.6, 1.1.1.86, 4.2.1.9, 2.6.1.42]
rn:U0990	false	2-(A-Hydroxyethyl)thiamine_diP + NADPH + PYR = 3-Methyl-2-ox-obutanoate + NADP+ + Thiamin_diP	EZComp_Val_Meta [2.2.1.6, 1.1.1.86, 4.2.1.9]
rn:U0991	false	3-Methyl-2-oxobutanoate + Ac-CoA + L-Glu + NAD+ = AKG + CoA + L-Leu + NADH	EZComp_Leu_Meta [2.3.3.13, 4.2.1.33, 1.1.1.85, 2.6.1.42]
rn:U0993	false	L-Asp + L-Citrulline = Fum + L-Arg	EZComp_Fum_Asp [4.3.2.1, 6.3.4.5]
rn:U0994	false	CoA + NAD+ + PYR = Ac-CoA + NADH	EZComp_PyrAcetylCoA [1.2.4.1, 2.3.1.12, 1.8.1.4]
rn:U0995	false	Gly + NAD+ + THF = 510-MethyleneTHF + NADH + NH3	EZComp_GlySerThrMeta [2.1.2.10, 1.8.1.4, 1.4.4.2]
rn:U0996	false	Ac-CoA + L-Cys + L-HomoSer = Ac + CoA + L-HomoCys + NH3 + PYR	EZComp_MetMeta [2.3.1.31, 2.5.1.48, 4.4.1.8]
rn:U0997	false	AKG + L-Orn + NADH + 2 NADPH = 2 L-Pro + NAD+ + 2 NADP+	EZComp_UreaMeta [1.5.1.2, 1.5.1.12, 2.6.1.13]

Table 15: *S. epidermidis* network - Elementary Modes discussed in the results section[1]

#	Flux sum	Rev.?[2]	Length	Reactions[3]	Net reaction
16	27	false	22	(1 rn:E0019) (1 rn:N02) (1 rn:U0038) (1 rn:U0061) (1 rn:U0195) (2 rn:U0238) (1 rn:U0239) (2 rn:U0255) (2 rn:U0256) (1 rn:U0260) (2 rn:U0270) (1 rn:U0276) (1 rn:U0280) (1 rn:U0297) (1 rn:U0301) (1 rn:U0302) (1 rn:U0311) (1 rn:U0360) (1 rn:U0372) (1 rn:U0522) (2 rn:U0534) (1 rn:U0990)	4 D-G(ex) + 6 NAD+ + 5 NH3(ex) = 2 Ala-tRNA(ex) + 6 NADH + Phe-tRNA(ex) + 2 Ser-tRNA(ex)
17	148	false	25	(6 rn:E0019) (6 rn:U0038) (6 rn:U0061) (6 rn:U0195) (12 rn:U0238) (5 rn:U0239) (12 rn:U0255) (12 rn:U0256) (6 rn:U0260) (5 rn:U0269) (12 rn:U0270) (5 rn:U0276) (-2 rn:U0295) (6 rn:U0297) (6 rn:U0301) (1 rn:U0302) (2 rn:U0311) (4 rn:U0314) (2 rn:U0315) (-2 rn:U0316) (5 rn:U0360) (6 rn:U0372) (6 rn:U0522) (7 rn:U0534) (6 rn:U0990)	7 D-G(ex) + 12 NAD+ + 6 NH3(ex) = 12 NADH + 6 Val-tRNA(ex)
18	221	false	30	(5 rn:E0019) (4 rn:N02) (5 rn:U0038) (15 rn:U0061) (10 rn:U0089) (10 rn:U0093) (5 rn:U0195) (10 rn:U0238) (4 rn:U0239) (10 rn:U0244) (10 rn:U0246) (10 rn:U0255) (10 rn:U0256) (10 rn:U0260) (10 rn:U0270) (4 rn:U0276) (4 rn:U0280) (-2 rn:U0295) (10 rn:U0297) (10 rn:U0301) (6 rn:U0311) (4 rn:U0314) (2 rn:U0315) (-2 rn:U0316) (4 rn:U0360) (15 rn:U0372) (5 rn:U0522) (10 rn:U0534) (5 rn:U0990) (10 rn:U0993)	2 D-G(ex) + 2 NAD+ + NH3(ex) = 2 NADH + Pyrimidine(ex) + Val-tRNA(ex)
22	294	false	34	(5 rn:E0012) (5 rn:E0018) (6 rn:N02) (5 rn:U0047) (5 rn:U0059) (15 rn:U0061) (5 rn:U0062) (10 rn:U0093) (-5 rn:U0106) (5 rn:U0121) (5 rn:U0138) (5 rn:U0195) (15 rn:U0238) (6 rn:U0239) (10 rn:U0244) (-10 rn:U0245) (15 rn:U0255) (15 rn:U0256) (15 rn:U0260) (15 rn:U0270) (6 rn:U0276) (6 rn:U0280) (-3 rn:U0295) (15 rn:U0297) (15 rn:U0301) (9 rn:U0311) (6 rn:U0314) (3 rn:U0315) (-3 rn:U0316) (6 rn:U0360) (15 rn:U0372) (10 rn:U0522) (15 rn:U0534) (5 rn:U0989)	9 D-G(ex) + 13 NAD+ + 11 NH3(ex) = 9 Cys-tRNA(ex) + 13 NADH + 2 Phe-tRNA(ex) + Pyrimidine(ex)

[1] *complete list availabe from the authors on request*

[2] *reversible*

[3] *reaction identifiers are taken from KEGG, for annotations see the reaction table*
